# PD‐L1 Expression in Acute Myeloid Leukemia Cells: Associations With Cell Metabolism

**DOI:** 10.1155/jimr/1427790

**Published:** 2026-07-01

**Authors:** Tereza Kořánová, Barbora Brodská, Antonín Ptáček, Petra Otevřelová, Marek Jedlička, Jan Musil, Zdenka Gašová, Jan Válka, Kateřina Kuželová

**Affiliations:** ^1^ Department of Proteomics, Institute of Hematology and Blood Transfusion, 12820, Prague, Czech Republic, uhkt.cz; ^2^ Department of Immunomonitoring and Flow Cytometry, Institute of Hematology and Blood Transfusion, 12820, Prague, Czech Republic, uhkt.cz; ^3^ Department of Modern Immunotherapy, Institute of Hematology and Blood Transfusion, 12820, Prague, Czech Republic, uhkt.cz; ^4^ Department of Apheresis, Institute of Hematology and Blood Transfusion, 12820, Prague, Czech Republic, uhkt.cz; ^5^ Clinical Department, Institute of Hematology and Blood Transfusion, 12820, Prague, Czech Republic, uhkt.cz

**Keywords:** cell metabolism, glycolysis, immune escape, PKM2-IN-1, Seahorse, stattic

## Abstract

The programmed death ligand 1 (PD‐L1) is a prominent mediator of immune system inhibition in various cancer types. In acute myeloid leukemia (AML), the prognostic meaning of PD‐L1 expression is still unclear and likely depends on the mechanism of its induction. We analyzed PD‐L1 expression (transcript and protein) in primary cells of patients with AML at diagnosis as a function of cell metabolic phenotype. The percentage of PD‐L1‐positive cells was typically low shortly after cell isolation but increased after overnight rest, in correlation with the cell glycolysis rate. The increase in PD‐L1 was prevented by pharmacological inhibition of the transcription factor STAT3 or pyruvate kinase M2 (PKM2) while JAK1/2 inhibition by ruxolitinib was less efficient. PD‐L1 positivity in freshly isolated cells was associated with increased levels of plasma IL‐6 and IL‐18. Furthermore, glycolytic primary cells induced PD‐L1 on cocultured AML cell lines. Although PD‐L1 was present at variable levels in exosomes released from primary cells, no correlation between the exosomal PD‐L1 and PD‐L1 on cocultured cells was observed. Our results suggest that PD‐L1 expression in leukemia cells is highly dynamic and regulated by PKM2/STAT3. Bulk AML cells can induce PD‐L1 on more primitive leukemia cells and support their immune evasion.

## 1. Introduction

Increasing evidence points to an important role of the immune system in leukemia prevention and eradication. Immune profiling at various disease states might help to predict relapses resulting from immune evasion, but the prognostic relevance of immune escape markers has not been clearly established. Expression of inhibitory ligands to various T‐cell receptors belongs to the main mechanisms involved in immune escape, and high expression of the programmed death ligand 1 (PD‐L1) has been associated with worse outcomes in acute myeloid leukemia (AML) [1–3].

In general, factors affecting activation of immune escape mechanisms can be cell‐inherent [4, 5] or external, the latter involving both soluble factors and contact with other cells. Certain recurrent genetic aberrations, such as the JAK2 V617F mutation or NPM‐ALK fusion, lead to constitutive PD‐L1 expression on cancer cells. Such cell‐inherent mechanisms were described in AML with TP53 mutation [6] or aberrant JAK2/STAT signaling [7]. Known extrinsic factors inducing PD‐L1 on AML cells include cytokines, such as IFNγ or TNFα, and ligands to Toll‐like receptors [8, 9].

In addition, PD‐L1 expression was related to cell glycolytic activity in various tumor cell types [10–13]. Although healthy hematopoietic stem cells use glycolysis as the main metabolic pathway, their leukemia counterparts highly depend on oxidative phosphorylation [14, 15]. The metabolic phenotype of AML cells has been proposed as an independent prognostic factor in AML [16, 17]. We thus aimed to explore the possible relation between metabolic activity and the PD‐L1 surface amount in primary cells from patients with AML at diagnosis.

## 2. Materials and Methods

### 2.1. Cell Isolation and Culture

Primary cells from the peripheral blood of AML patients (*N* = 45) were obtained by apheresis before chemotherapy initiation. The leukapheretic product was diluted 10‐fold in phosphate‐buffered saline (PBS), and mononuclear cells (PBMCs) were separated using Histopaque‐1077 (Sigma‐Aldrich, #H8889). PBMCs were washed three times in PBS and resuspended at 5 × 10^6^/mL in RPMI‐1640 medium with 10% fetal calf serum (FCS), 2 mM L‐alanyl‐L‐glutamine, 100 U/mL penicillin, and 100 µg/mL streptomycin. If not otherwise specified, the experiments were performed using freshly isolated cells. Sample aliquots were cryopreserved immediately after cell isolation and used to analyze the effect of inhibitors or glucose‐free media.

In a control unselected cohort of patients with AML at diagnosis (*N* = 22), PBMC were isolated from peripheral blood samples (2 mL of EDTA‐anticoagulated whole blood) using Histopaque‐1077, washed in PBS, and analyzed by flow cytometry using the same protocol as the cells obtained from leukapheresis.

KG‐1 cells (RRID:CVCL_0374) established from human male bone marrow were obtained from the German Collection of Microorganisms (DSMZ) in 2017. THP‐1 cells (RRID:CVCL_0006) established from human male peripheral blood were purchased from Merck Life Science in 2021. The cell lines were not further authenticated for the described experiments. No misidentification or contamination of these cell lines was reported according to the ICLAC Register of Misidentified Cell Lines version 13. The cell cultures were regularly confirmed as free from mycoplasma contamination by qPCR. Leukemia cell lines were cultured in culture medium with FCS, glutamine, and antibiotics under standard conditions (5% CO_2_, 37°C), as recommended by the suppliers. For coculture experiments, cell lines were stained with carboxyfluorescein succinimidyl ester (CFSE) (Cell Division Tracker kit, BioLegend, #423801) or CellTrace Far Red (Invitrogen, #C34564) and resuspended in fresh RPMI‐1640 at 1 × 10^6^/mL. Aliquots were mixed 1:1 with conditioned media (CMs) from the cell line (control), CM from primary AML cells, or primary cell suspension (5 × 10^6^/mL).

### 2.2. Chemicals

Stattic (Sigma, S7947‐25 MG) stock solution was prepared at a 5 mM concentration in dimethyl sulfoxide (DMSO), ruxolitinib (MedChemExpress, HY‐50856) at 100 µM in DMSO, and PKM2‐IN‐1 (MedChemExpress, HY‐103617) at 10 mM in DMSO. Working solutions were made by diluting the stock solutions in RPMI‐1640 medium.

### 2.3. Flow Cytometry

Sample aliquots were washed once in PBS and stained with CD45‐Pacific blue (BD Biosciences, #560367) alone or in combination with PD‐L1‐PE (Exbio, #1P‐177‐T100) for 30 min at 5°C. In a limited number of samples, CD34‐AF647 (BioLegend, #343508) and CD38‐BV786 (BD Biosciences, #563964) were added to distinguish cell subpopulations with different maturity degrees. For cryopreserved cells, human immunoglobulins (IgG, final concentration: 0.1 mg/mL) were added along with the antibodies to block unspecific binding. Cells were washed once in PBS and analyzed using a BD LSRFortessa or BD FACSymphony flow cytometer.

The gating strategy is shown in Supporting Information 1: Figure S1. Cell debris was gated out in the scattergrams. AML blasts were gated in CD45 versus SSC dotplots. Propidium iodide or 4,6‐diamidino‐2‐phenylindole (DAPI) were used to discriminate dead cells. Cell doublets were removed using FSC‐A versus FSC‐H dotplots. Cell lines in coculture were gated according to CFSE/CellTrace Far Red positivity. Fluorescence minus one (FMO) controls without the PD‐L1 antibody were used to determine the cutoff for PD‐L1 positivity and the background mean fluorescence intensity (MFI) value, which was subtracted from the MFI of the assay samples.

To sort primary cell subpopulations, a cell aliquot containing 20 × 10^6^ cells was thawed, washed in staining buffer containing human IgG, and stained for 30 min with CD45‐BUV805 (BD Biosciences, #612891), CD34‐AF647 (BioLegend, #343508), and CD38‐BV480 (BD Biosciences, #566137). DAPI was added prior to cell sorting using a BD FACSDiscover S8 device.

### 2.4. Metabolic Measurement

The oxygen consumption rate (OCR) and the extracellular acidification rate (ECAR) were measured using a Seahorse XFp device and the MitoStress Test kit (Agilent, #103010‐100), as described previously [18]. CellTak (Corning, #354240) was used to coat Seahorse XFp plates according to the manufacturer’s instructions. Leukemia cells (200,000 cells/well) were seeded in 50 µL of Seahorse medium (Agilent #103576‐100) with 2 mM glutamine, 10 mM glucose, and 1 mM pyruvate. The last injection of inhibitors was supplemented with 2‐deoxyglucose (Sigma, D8375) to obtain ECAR background values due to nonglycolytic acidification. The final well concentrations of the inhibitors were the following: oligomycin 2 µM, carbonyl cyanide‐4 (trifluoromethoxy) phenylhydrazone (FCCP) 0.6 and 1 µM, rotenone/antimycin A 0.5 µM, and 2‐deoxyglucose 50 mM.

### 2.5. Assessment of PD‐L1 Transcript Levels

RNA was isolated from 2 × 10^7^ cells using the RNeasy Mini kit (Qiagen #74104), and cDNA was obtained by reverse transcription (SensiFAST cDNA Synthesis Kit, Bioline, #BIO65054). Relative amounts of the PD‐L1 transcript were calculated from the expression ratio of PD‐L1 and GAPDH qPCR products measured using the SensiFAST SYBR No‐ROX Kit (Bioline #BIO98020). The respective forward and reverse primer sequences were: ATGGTGGTGCCGACTACAAG and GGAATTGGTGGTGGTGGTCT for PD‐L1, GAAACTGTGGCGTGATGGC and CCGTTCAGCTCAGGGATGAC for GAPDH.

### 2.6. Western Blot Analysis of PKM2 and Phospho‐STAT3 Protein Amounts

Protein lysates were prepared from freshly isolated PBMC. Cells (2 × 10^8^) were washed once with ice‐cold PBS, resuspended in 1 mL of 2× sample buffer (SB) (100 mM Tris, HCl pH 6.8, 4% SDS, 200 mM DTT, and 20% glycerol), incubated for 5 min at 95°C, and spun using a Sorvall WX + ultracentrifuge (4 h; 180,000 × *g*; 4°C). The supernatant was stored at −80°C until analysis by western blot. Protein samples were resolved in 7.5% polyacrylamide gel (10 cm× 6 cm) and transferred to a nitrocellulose membrane using the Trans‐Blot Turbo transfer system. The membrane was blocked for 1 h in 3% bovine serum albumin and incubated overnight at 5°C with PKM2 antibody (Cell Signaling Technology, #4053) diluted 1:4000 in Tris‐buffered saline with 0.1% Tween‐20 (TBST) or pSTAT3/STAT3 antibody (Cell Signaling Technology, 9145S/30835S) diluted 1:2000 in TBST. The membrane was washed six times with TBST, incubated for 1 h with horseradish peroxidase‐conjugated secondary antibody (1:10,000 for PKM2, 1: 20,000 for pSTAT3/STAT3), and washed again six times with TBST. The chemiluminescence signal of Clarity Western ECL Substrate (Bio‐Rad, #170−5060) was detected using a G:BOX Chemi XX6 device (Syngene).

### 2.7. Analysis of Inflammatory Molecules in AML Plasma

The levels of inflammatory cytokines (IL‐1β, IFNα2, IFNγ, TNFα, MCP‐1, CXCL‐8, IL‐6, IL‐10, IL12p70, IL17A, IL‐18, IL‐23, and IL‐33) in the plasma of AML patients (*N* = 17) were quantified using the LEGENDlex Human Inflammation Panel 1 (Biolegend, #740809) according to the manufacturer‘s instructions. Briefly, thawed plasma samples were centrifuged (800 *g*; 5 min) to remove cell debris. Subsequently, undiluted plasma samples were incubated for 2 h with premixed antibody‐coated beads on a microplate shaker. After washing, detection antibodies were added, and the mixture was incubated with shaking for 1 h. Finally, streptavidin‐phycoerythrin was added, and the mixture was incubated with shaking for additional 30 min. Samples were acquired using a Cytek Aurora flow cytometer, and FCS files were analyzed using LEGENDplex Data Analysis Software Suite (Qognit).

### 2.8. Exosome Preparation and Analysis

PBMC were cultured for 24–48 h (resp. 5 days for samples E1 and E2) in RPMI‐1640 medium with 10% exosome‐depleted FCS. Exosomes were isolated from 400 mL of cell culture media, as previously described [19]. Briefly, two‐step centrifugation was used to remove the cells, and the supernatant was filtered to remove larger vesicles and cell debris. The resulting suspension was spun, exosome pellets were resuspended in PBS, collected in one tube, and sedimented again. The final pellet was resuspended in PBS, mixed 1:1 with 2× SB, and heated for 10 min at 95°C. To obtain the corresponding source cell sample, a part of the first centrifugation pellet was washed with PBS, lysed in a lysis buffer (10 mM Tris HCl pH 7.5, 150 mM NaCl, 0.5 mM EDTA, 0.5% NP‐40, with freshly added protease and phosphatase inhibitors) for 30 min at 4°C, and centrifuged (10 min; 20,000 *g*; 4°C). The supernatant was mixed with 2× SB and heated for 10 min at 95°C. All samples were stored at −20°C until use. SDS‐PAGE (12% acrylamide) followed by immunoblotting (PVDF membrane, Bio‐Rad) was performed to assess the protein levels. Mouse antibodies against PD‐L1 (BD Pharmingen, #557924, or Santa Cruz Biotechnology, sc‐293425), CD81 (Santa Cruz Biotechnology, sc‐166029), and β‐actin (Santa Cruz Biotechnology, sc‐47778) were visualized by HRP‐conjugated goat anti‐mouse antibody (ThermoFisher Scientific, #31430). The chemiluminescence signal from Clarity Western ECL Substrate (Bio‐Rad, #170−5061) or Westar Supernova (Cyanagen, #XLS3,0100) was acquired and evaluated by G:box Chemi XX6 device (Syngene). Precision Plus Protein All Blue Protein Standards (BioRad, #161‐0373) or Precision Plus Protein^TM^ Dual Color Protein Standards (BioRad, #161‐0374) were used to determine the molecular weight of the western blot bands.

### 2.9. Statistics

Statistical evaluation was performed using GraphPad Prism version 9.5.0 (GraphPad Software, San Diego, California, USA). The *p*‐value limit for statistically significant differences was set to 0.05. The Shapiro–Wilk test was used to evaluate data normality and parametric or nonparametric tests were used accordingly.

## 3. Results

**Figure 1:**
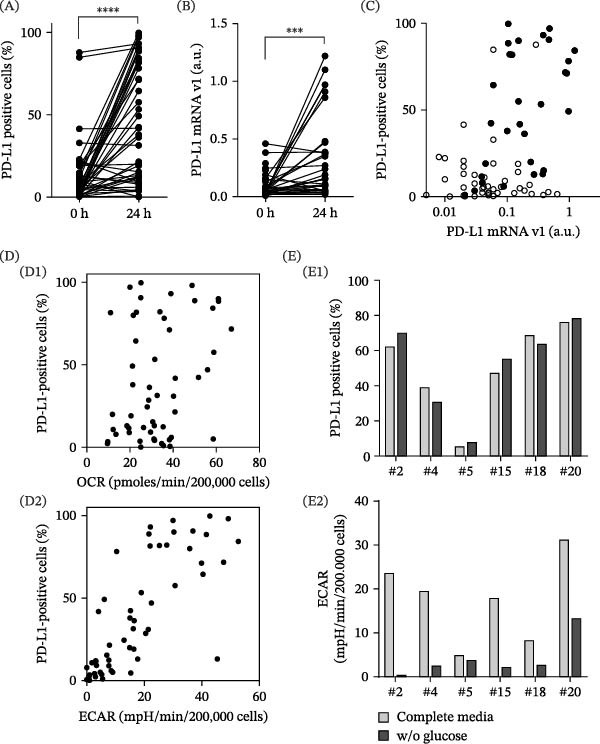
Changes in PD‐L1 expression during in vitro culture of primary AML cells. PBMC from patients with AML at diagnosis (*N* = 45) were isolated from leukapheretic samples and cultured overnight. PD‐L1 was tested immediately after isolation (0 h) and after overnight rest (24 h), both at the surface protein (A) and transcript (B) levels. The difference between 0 h and 24 h (A, B) was evaluated using a paired Student’s *t*‐test,  ^∗∗∗^ *p* < 0.001,  ^∗∗∗∗^ *p* < 0.0001. (C) Correlation between PD‐L1 surface protein and transcript (empty symbols: 0 h, closed symbols: 24 h). Spearman correlation test: *r* = −0.09138, *p* = 0.5601 for 0 h; *r* = 0.4836, *p* = 0.0068 for 24 h data. (D) Correlation of PD‐L1 positivity with primary cell metabolic activity. The fraction of PD‐L1‐positive cells determined after 24 h of in vitro culture is plotted against the cellular respiration rate (OCR, D1) and glycolysis rate (ECAR, D2). Spearman correlation test: OCR *r* = 0.26, *p* = 0.0576; ECAR *r* = 0.8242, *p* < 0.0001. (E) PD‐L1 induction in glucose‐free medium. Frozen samples from six different individuals were thawed and cultured overnight in complete or glucose‐free medium. PD‐L1 expression was measured by flow cytometry on gated viable cells (E1). Sample aliquots were used to measure ECAR (E2). Paired Student’s *t*‐test: *p* = 0.0109 for ECAR, *p* = 0.6799 for PD‐L1.

The clinical and genetic data corresponding to the cohort of AML samples from leukapheresis (*N* = 45) is provided in the Supporting Information 2: Table S1. Surface PD‐L1 expression was measured by flow cytometry immediately after PBMC isolation (0 h time point) and after an overnight in vitro culture (24 h time point). As shown in Figure 1A, the percentage of PD‐L1‐positive cells increased during incubation in 60% of samples (27 of 45). Similar results were obtained when the MFI was used to quantify PD‐L1 levels (Supporting Information 1: Figure S2A). The PD‐L1 transcript was measured by real‐time qPCR. The number of samples available for mRNA analysis at the 24 h timepoint was lower (*N* = 32), but the trend was similar: the full‐length PD‐L1 transcript increased in 66% of samples (21 of 32) during the overnight rest (Figure 1B). In line with our previous report [1], surface PD‐L1 protein did not significantly correlate with the full‐length transcript in freshly isolated cells (Figure 1C, empty symbols). In contrast, a positive correlation was observed after 24 h of incubation (closed symbols, Spearman correlation test: *r* = 0.4836, *p* = 0.0068).

Leukapheresis is performed as a part of AML therapy in a subset of patients with hyperleukocytosis, that is very high white blood cell counts (WBC > 100 × 10^9^/L). To investigate the potential association between PD‐L1 expression and hyperleukocytosis, PD‐L1 surface levels were also measured in an unselected control cohort using PBMC isolated from whole blood samples (*N* = 22). No substantial difference in PD‐L1 positivity between samples from leukapheresis and unselected cohort was detected (Supporting Information 1: Figure S3; Mann–Whitney test: *p* = 0.1651).

To check for possible AML cell activation by FCS used in the culture medium, some samples were cultured in parallel in RPMI‐1640 with human serum (HS). The amount of surface PD‐L1 was actually lower in cells with HS, but a large PD‐L1 positivity due to overnight culture was consistently detected regardless of the serum type (Supporting Information 1: Figure S4).

The cell respiration rate (OCR) and glycolysis rate (ECAR) in cells obtained from leukapheresis were determined using the Seahorse platform. In preliminary experiments, the cell metabolic activity was very low immediately after PBMC isolation. The metabolic measurements were thus only performed after 24 h of culture. The type of serum (HS vs. FCS) in culture media had no significant effect on the cell metabolism (Supporting Information 1: Figure S4). PD‐L1 positivity strongly correlated with ECAR (Figure 1D and Supporting Information 1: Figure S2B). PD‐L1 correlation with OCR was less pronounced and did not reach statistical significance for the PD‐L1‐positive cell fraction (Figure 1D).

The possible causative link between cell glycolytic activity and PD‐L1 expression was analyzed by in vitro culture in a glucose‐free medium. Cryopreserved aliquots of selected samples, frozen immediately after cell isolation, were used to compare PD‐L1 evolution in the media with and without glucose. PD‐L1 levels were very low shortly after cell thawing (less than 1% of positive cells) and markedly increased upon overnight rest. Surprisingly, no difference between samples incubated with and without glucose was found, despite ECAR reduction in the absence of glucose (Figure 1E and Supporting Information 1: Figure S2C). Statistical analysis (paired Student’s *t*‐test) confirmed significant effect of glucose starvation on ECAR (*p* = 0.0109), but not on PD‐L1 expression (*p* = 0.6799).

Signal transducer and activator of transcription 3 (STAT3) regulates PD‐L1 transcription and cell metabolism. We thus tested the effect of STAT3 inhibition on the observed in vitro PD‐L1 dynamics. Cryopreserved primary cells were thawed and incubated overnight in the presence or absence of the STAT3 inhibitor stattic. Stattic at a 5 µM concentration at least partly prevented PD‐L1 induction and reduced ECAR (Supporting Information 1: Figure S5). As expected, STAT3 inhibition also largely impaired cellular respiration (OCR).

**Figure 2:**
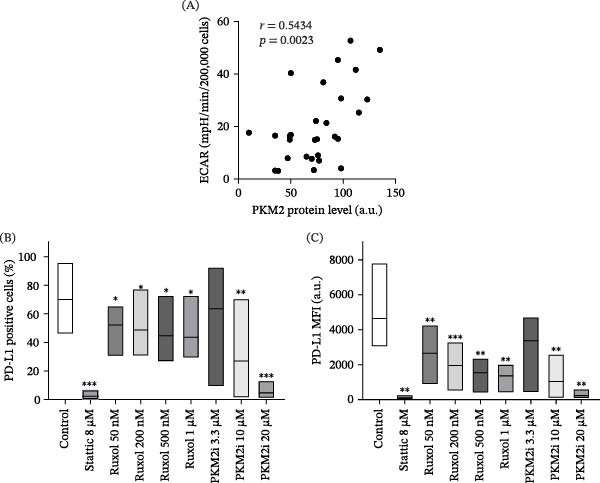
Importance of PKM2 for glycolysis and PD‐L1 induction in primary AML cells. (A) PKM2 protein levels in AML primary cells (*N* = 29) were determined by western blot and plotted against the glycolysis rate (ECAR). Examples of western blot membranes are given in Supporting Information 1: Figure S6A. Correlation was assessed using the Pearson correlation test, *r* = 0.5434, *p* = 0.0023. (B + C) Effect of STAT3/JAK/PKM2 inhibition on PD‐L1 induction during in vitro culture of primary AML cells (*N* = 7). Cryopreserved aliquots of primary cells were thawed, treated with inhibitors as indicated (stattic; ruxol: ruxolitinib; PKM2i: PKM2‐IN‐1), and cultured overnight. Surface PD‐L1 on gated viable AML cells was analyzed by flow cytometry and quantified as the fraction of PD‐L1 positive cells (B) or mean fluorescence intensity (MFI, C). The floating bars show the means and extreme values from seven independent experiments. Differences between treated cells and the corresponding controls were evaluated using the paired Student’s *t*‐test ( ^∗^ *p* < 0.05,  ^∗∗^ *p* < 0.01,  ^∗∗∗^ *p* < 0.001).

STAT3 is canonically activated by Janus kinases (JAK1/2), but its function in gene transcription can also be enhanced by other kinases. Notably, the glycolytic enzyme pyruvate kinase M2 (PKM2) regulates PD‐L1 expression in macrophages, dendritic cells, and T cells [20]. As shown in Figure 2A and Supporting Information 1: Figure S6A, PKM2 was expressed in primary AML cells, and its relative content correlated with ECAR (Pearson correlation coefficient *r* = 0.5434, *p* = 0.0023). The impact of JAK1/2 or PKM2 inhibition on PD‐L1 induction was analyzed in comparison with 8 µM stattic in cells with high PD‐L1 levels. These cells usually displayed high STAT3 phosphorylation, although one sample only had JAK2 V617F mutation (Supporting Information 1: Figure S6B). Ruxolitinib reduced the PD‐L1‐positive cell fraction and PD‐L1 MFI less efficiently compared to PKM2 or STAT3 inhibition (Figure 2B,C). Western blot analysis confirmed that the ruxolitinib dose was sufficient to achieve the maximal effect on STAT3 phosphorylation (Supporting Information 1: Figure S7A). The superiority of stattic and PKM2‐IN‐1 over ruxolitinib was observed even in the sample with JAK2 V617F (Supporting Information 1: Figure S7B). The effect of PKM2‐IN‐1 on the cell metabolic rates was similar to that of stattic (Supporting Information 1: Figures S5B and S8).

**Figure 3:**
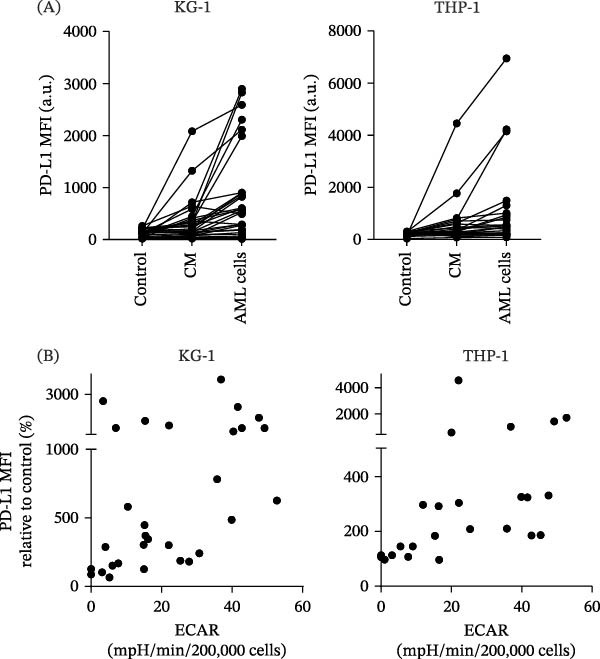
Primary AML cells induce PD‐L1 expression on AML cell lines. (A) Stained KG‐1 or THP‐1 cells were cultured overnight in RPMI‐1640 medium (control), conditioned medium from primary AML cells (CM) or with primary AML cells (*N* = 31 for KG‐1, 25 for THP‐1). The graph shows the mean fluorescence intensity (MFI) of the PD‐L1‐PE antibody from gated viable KG‐1/THP‐1 cells. (B) PD‐L1 increase induced by coculture with primary AML cells was normalized using the corresponding controls (100%) and plotted as a function of the primary cell glycolytic rate (ECAR). Spearman correlation test: *r* = 0.5558, *p* = 0.0014 for KG‐1; *r* = 0.7237, *p* < 0.0001 for THP‐1.

To assess possible paracrine effects, coculture experiments were performed with AML cell lines KG‐1 and THP‐1, which have low but reliably detectable basal PD‐L1 expression. Primary AML cells were cultured for at least 24 h after isolation prior to their use in coculture experiments. Cell lines were prestained and incubated overnight with CM from primary cells or with primary cells. PD‐L1 MFI from gated viable KG‐1/THP‐1 cells in these samples was compared with the MFI from the control cells incubated in RPMI‐1640. Primary AML cells induced a variable PD‐L1 increase in cocultured cells (Figure 3A). This increase correlated with ECAR of primary cells (Figure 3B, Spearman’s *r* = 0.5558, *p* = 0.0014 for KG‐1; *r* = 0.7237, *p* < 0.0001 for THP‐1).

**Figure 4:**
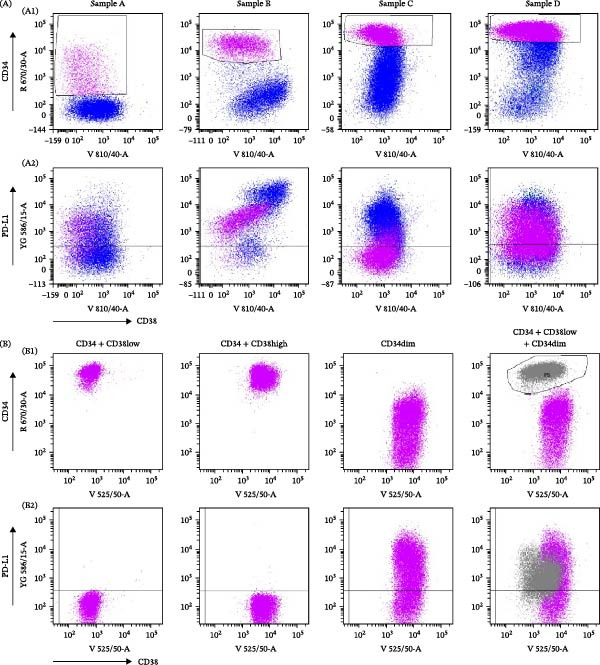
PD‐L1 induction in CD34/CD38 gated subpopulations. (A) Frozen aliquots of four different primary AML samples were thawed, cultured overnight, and stained for CD34, CD38, and PD‐L1. Viable cells with the highest CD34 expression were gated in CD34 versus CD38 dotplots (A1) and shown in magenta. PD‐L1 was displayed against CD38 using the same color code (A2). (B) Another aliquot of sample D was thawed and sorted to obtain three subpopulations with different CD34/CD38 positivity as described. The subpopulations were cultured overnight individually or as a mixture. The cells were restained using the same antibodies as for cell sorting and analyzed by flow cytometry.

Leukapheretic samples are cell mixtures with variable differentiation degrees. To evaluate possible differences between immature leukemia progenitors and more differentiated cell forms, four samples previously found as PD‐L1‐positive were reanalyzed using CD34 and CD38 antibodies in addition to PD‐L1. Figure 4A shows the distribution of CD34 and CD38 in each individual sample (Figure 4A1) and PD‐L1 expression in cell subpopulations gated according to the CD34 expression level (Figure 4A2). Except for sample C, even the most immature cells (CD34high and CD38low) were largely positive in PD‐L1 after overnight culture. To distinguish between autocrine and paracrine PD‐L1 induction, another aliquot of sample D was thawed and sorted into three subpopulations: the less mature CD34high CD38low, intermediate CD34high CD38high, and the most differentiated CD34dim. The subpopulations were then cultured overnight in separate wells. In parallel, aliquots of the two extreme populations were mixed and cocultured. As shown in Figure 4B, PD‐L1 was not induced in CD34high cells cultured separately, regardless of the CD38 amount, while the positivity of CD34dim cells corresponded to the previous results shown in Figure 4A (sample D). However, CD34high CD38low cells did induce PD‐L1 in coculture with CD34dim cells (gray subpopulation in Figure 4B). This indicates that PD‐L1 induction is autocrine in differentiated cells but paracrine in stem‐like cells.

PD‐L1 was also induced by CM, although less efficiently than in coculture with primary cells (Figure 3A). This finding points to the role of secreted compounds, which could include glycolytic metabolites, proinflammatory molecules, or even PD‐L1 itself, either secreted in a soluble form or as a part of exosomes released from primary cells. Lactate was previously shown to stimulate PD‐L1 expression on AML cells, but we detected no significant change in the PD‐L1 surface protein after cell treatment with lactate (Supporting Information 1: Figure S9).

**Figure 5:**
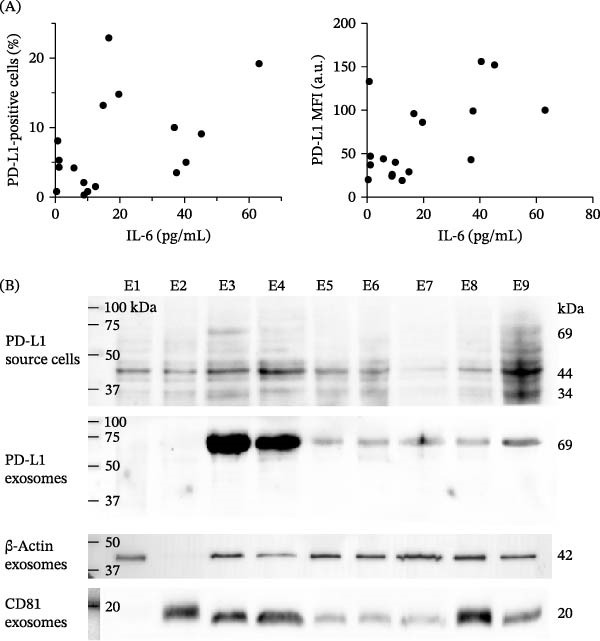
Analysis of plasma cytokines and exosomal PD‐L1. (A) Inflammatory cytokines were measured in the plasma of 17 AML patients at diagnosis and their amounts were correlated with surface PD‐L1 of AML primary cells in the matched samples. All results of statistical analyses are given in Table 1. The graphs show the results obtained for IL‐6. (B) PD‐L1 in AML exosomes and source primary AML cells was detected by western blot. CD81 was used as a standard exosomal marker. The figure shows examples of western blot membranes. Correlations of the exosomal PD‐L1 content normalized to β‐actin with selected measured parameters are shown in Supporting Information 1: Figure S11.

Plasma samples cryopreserved at AML diagnosis were available for 17 of 45 patients included in our cohort. Inflammatory molecules in the plasma were measured using flow cytometry and correlated with PD‐L1 surface amounts in the matched samples of primary cells (Table 1). While no correlation was found for the majority of cytokines and chemokines, the analysis suggested an association of PD‐L1 levels on AML cells with higher plasma IL‐6 (Figure 5A) and IL‐18 (Supporting Information 1: Figure S10).

**Table 1:** Correlation of surface PD‐L1 with inflammatory cytokines and chemokines in AML plasma.

Cytokine	PD‐L1 positive cell fraction		PD‐L1 MFI	
	Spearman’s *r*	*p*‐Value	Spearman’s *r*	*p*‐Value
IL‐1β	0.0615	0.8138	−0.059	0.8216
IFNα2	0.06871	0.7922	0.01717	0.949
IFNγ	0.03938	0.8803	0.08487	0.7448
TNFα	0.1513	0.559	−0.03196	0.9036
MCP‐1	0.1247	0.6304	0.1964	0.4469
IL‐6	**0.4847**	**0.0503**	**0.5297**	** ^∗^0.0305**
CXCL8	0.185	0.4740	0.106	0.6838
IL‐10	0.1509	0.5601	−0.1349	0.6033
IL‐12p70	0.3067	0.2293	−0.02943	0.9114
IL‐17A	0.2908	0.2554	0.1668	0.5198
IL‐18	**0.5641**	** ^∗^0.0200**	0.2010	0.4378
IL‐23	0.4304	0.0857	0.1348	0.6053
IL‐33	0.0384	0.8848	−0.06867	0.7927

*Note:* Inflammatory molecules were quantified using the LegendPlex Human Inflammation Panel 1 in the plasma of AML patients (*N* = 17) and correlated with surface PD‐L1 at the 0 h time point in matched leukapheretic samples. The positive cell fraction or mean fluorescence intensity (MFI) was alternatively used to quantify PD‐L1 levels. Results with statistically significant or borderline differences are highlighted in bold.

^∗^Statistically significant correlation at the 0.05 level.

PD‐L1 amounts in exosomes produced by AML cells were tested by western blot in 16 samples. PD‐L1 was detected in all AML cell lysates, the dominant band being situated at 44 kDa (Figure 5B). In contrast, the exosomal PD‐L1 amount was more variable, and the band was shifted to a higher molecular weight (69 kDa). The specificity of the exosomal PD‐L1 band was confirmed by another antibody, showing a band at the same position. However, no obvious correlation of the exosomal PD‐L1 with ECAR, surface PD‐L1 on primary cells, or PD‐L1 induction on KG‐1 cells was noted (Supporting Information 1: Figure S11).

## 4. Discussion

Analysis of primary cells obtained from patients with AML at diagnosis showed that the PD‐L1 expression on AML blasts is highly dynamic (Figure 1A, B). Large changes in the PD‐L1 amount during the overnight cell rest, a common part of protocols for PBMC analysis by flow cytometry [21], may contribute to the variability in reported prognostic implications of PD‐L1 positivity in AML.

As reported previously, the PD‐L1 surface protein does not correlate with the full‐length transcript (mRNA variant 1, NM_014143.4) in AML cells [1]. This can be due to a variety of mechanisms involved in PD‐L1 processing and surface exposition. Similar to this work, PD‐L1 protein expression on freshly isolated AML cells did not correlate with the transcript (Figure 1C, empty symbols). Nevertheless, the increase in PD‐L1 surface exposition during the overnight rest was at least partly mediated by increased transcript levels, as suggested by transcript versus protein correlation at the 24 h time point (Figure 1C, closed symbols).

PD‐L1 expression changes correlated with cell metabolism, notably ECAR (Figure 1D, Supporting Information 1: Figure S2B). However, they were independent of glucose in the culture medium (Figure 1E, Supporting Information 1: Figure S2C). The observed changes in PD‐L1 amounts were thus related to the cell metabolic settings rather than to the actual metabolic activity. The correlation between PD‐L1 induction and cell metabolism might result from shared regulatory elements. A possible connection point between these processes is STAT3, a prominent regulator of PD‐L1 expression in hematological diseases [7, 22–24], which also modulates cell metabolism [25–27]. PD‐L1 induction was indeed prevented by the STAT3 inhibitor stattic (Supporting Information 1: Figure S5 and Figure 2B,C). STAT3 is canonically activated via JAK1/2, and the JAK2 V617F variant with constitutive kinase activity promotes PD‐L1 surface expression [23]. JAK1/2 inhibition by ruxolitinib actually reduced PD‐L1 levels, but the effect was lower compared to that of direct STAT3 inhibition by stattic (Figure 2B,C), even in the sample with JAK2 V617F (Supporting Information 1: Figure S7B).

**Figure 6:**
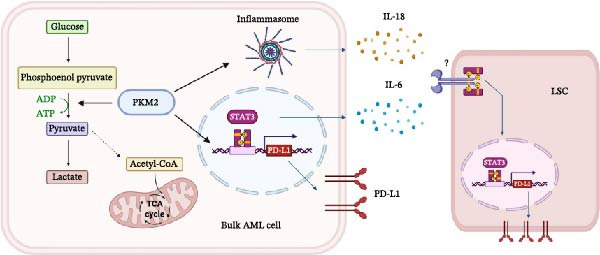
Scheme of the proposed regulatory mechanisms of PD‐L1 expression in AML cells. The pyruvate kinase M2 (PKM2) catalyzes conversion of phosphoenolpyruvate (PEP) to pyruvate in the glycolytic pathway. In parallel, the nuclear form of PKM2 promotes STAT3 phosphorylation in bulk AML cells. Increased STAT3 activity results in increased transcription of PD‐L1. PKM2‐mediated secretion of proinflammatory cytokines like IL‐6 and IL‐18 induces PD‐L1 in more primitive AML cells. LSC, leukemia stem cell; TCA cycle, tricarboxylic acid cycle.

Another link between PD‐L1 and cell metabolism is provided by PKM2, which regulates PD‐L1 expression via direct binding to the PD‐L1 promoter in human immune and cancer cells [20]. PKM2 is one of four pyruvate kinase isozymes converting phosphoenolpyruvate (PEP) to pyruvate in the glycolytic pathway. The PKM2 splicing variant produced from the PKM gene is associated with cancer development and progression. PKM2 can form dimers with low affinity to PEP or highly active tetramers [28]. Besides its canonical metabolic function, PKM2 has other tumor‐supporting roles, including the regulation of gene transcription [29]. Dimeric PKM2 translocates to the nucleus and phosphorylates STAT3, enhancing its transcription activity [30]. Our experiments showed a positive correlation between PKM2 content in AML primary cells and their glycolytic activity (Figure 2A), confirming involvement of PKM2 in glycolysis. PD‐L1 induction was largely prevented by PKM2 inhibition (Figure 2B,C), and the effect of PKM2‐IN‐1 on the cell metabolism was similar to that of stattic (Supporting Information 1: Figures S5 and S8). However, PD‐L1 levels were not affected by the absence of glucose in the culture medium (Figure 1E). PKM2 thus emerges as a key regulator of PD‐L1 expression in glycolytic primary AML cells independently of its enzymatic role in the glycolytic pathway (Figure 6).

Cells with a higher ECAR were also more efficient in inducing PD‐L1 on cocultured cell lines (Figure 3B). This effect could be mediated by lactate, which was shown to increase PD‐L1 expression on AML cells via histone lactylation [31]. Nevertheless, cell treatment with lactate did not increase PD‐L1 expression (Supporting Information 1: Figure S9), and PD‐L1 upregulation is thus probably independent of lactate production by primary cells.

Increased STAT3 transcriptional activity due to STAT3 phosphorylation by PKM2 can result in enhanced IL‐6 production [30]. Consistently, higher plasma IL‐6 amounts were associated with a higher PD‐L1 expression on AML cells at diagnosis (Table 1, Figure 5A). IL‐6 is a key proinflammatory cytokine produced by AML cells. It promotes leukemogenesis by acting on the malignant cells and their microenvironment [32]. We speculate that IL‐6 secreted from bulk primary AML cells could contribute to the observed PD‐L1 induction on cocultured AML cell lines (Figure 3) or autologous CD34+ cells (Figure 4), possibly via JAK/STAT pathway activation (Figure 6). Furthermore, a statistically significant positive correlation with PD‐L1‐positive cell fraction was found for IL‐18 (Table 1), which can also be activated via PKM2 [33]. To our knowledge, no direct involvement of IL‐18 in PD‐L1 regulation has been reported so far. However, in vitro treatment of KG‐1 cells with IL‐18 led to increased production of IFNγ [34], which promotes PD‐L1 on leukemia cells [8, 9].

We assume that multiple mechanisms contribute to PD‐L1 expression in AML cells. Additional interconnection between cell metabolism and immune escape might be provided by the AMP‐activated protein kinase (AMPK), which operates in hypoxia or during nutrient starvation to switch the cell metabolism to an energy‐saving mode. In parallel, AMPK was reported to promote PD‐L1 protein degradation [35, 36]. In addition to crosstalk points resulting in at least partial coregulation of PD‐L1 expression and glycolysis, evidence for a direct impact of PD‐L1 expression on the cell metabolism was also reported: PD‐L1 overexpression in the MOLM‐13 cell line led to increased transcript levels of genes involved in glycolysis [37].

The exosomal PD‐L1 contributes to systemic immune suppression in various cancer types and strongly correlates with tumor staging [38]. We confirmed that PD‐L1 was present in exosomes released from primary AML cells (Figure 5B). As noted previously in B‐cell lymphoma [39], the exosomal PD‐L1 had a higher molecular weight compared to the form found in the source cells. The main PD‐L1 band was at 44 kDa in the source cells (Figure 5B). This band is dominant in leukemia cell lines with low PD‐L1 surface positivity [40]. PD‐L1 surface exposition is associated with the presence of additional bands at higher molecular weight, presumably due to more extensive glycosylation. The exosomal PD‐L1 thus probably corresponds to a highly glycosylated, membrane‐bound form with immunosuppressive capacity. However, the exosomal PD‐L1 content did not correlate with primary cells’ capacity to induce PD‐L1 on cocultured cells (Supporting Information 1: Figure S11).

Our experiments were conducted in vitro, but similar PD‐L1 dynamics might occur in vivo. AML cells could express more PD‐L1 when residing in the bone marrow due to hypoxia‐induced metabolic switch to glycolysis. In contrast to AML bulk cells, AML stem cells are metabolically more rigid and depend on oxidative phosphorylation [15]. Leukemia stem cells, mimicked by the cell lines in our coculture experiments (Figure 3) or by the more primitive CD34+ cell compartment in primary cell samples (Figure 4), might have higher PD‐L1 levels due to the presence of bulk leukemia cells. We speculate that reduction of the tumor mass by chemotherapy could result in reduced stimulation of residual leukemia stem cells by the bulk leukemia and thereby in reduced PD‐L1 expression on the residual leukemia cells. In this case, high PD‐L1 expression on AML cells at diagnosis would not necessarily imply immune system impairment in the remission phase of the disease. In contrast, PD‐L1 expression is likely to be retained on residual leukemia cells if it is induced by a cell‐inherent mechanism.

In addition to the canonical function consisting of inhibition of anti‐tumor T‐cells, PD‐L1 is involved in the intracellular signaling of cancer cells [41]. Depending on its localization, PD‐L1 may affect cancer cell growth, survival, or resistance to drugs. Although the relevance of these additional cancer‐supporting functions of PD‐L1 in AML cells is yet unknown, the observed PD‐L1 dynamic changes may reflect complex intracellular processes associated not only with increased resistance to the immune system but also with leukemia cell fitness and resistance to therapy.

## 5. Conclusions

PD‐L1 surface expression on primary AML cells is dynamic and associated with cell metabolic settings. PKM2 and STAT3 emerge as key regulatory elements of PD‐L1 expression in AML cells. Moreover, AML bulk leukemia cells can induce PD‐L1 on more primitive AML progenitors, contributing to their immune evasion and possibly to their fitness and resistance to therapy.

## Author Contributions

Conception and design: Kateřina Kuželová and Barbora Brodská. Investigation: Tereza Kořánová, Antonín Ptáček, Petra Otevřelová, Kateřina Kuželová, and Barbora Brodská. Analysis and interpretation of the data: Kateřina Kuželová, Tereza Kořánová, Barbora Brodská, and Jan Musil. Resources: Zdenka Gašová, Jan Válka, and Jan Musil. Writing – original draft: Kateřina Kuželová, Barbora Brodská, Tereza Kořánová, and Antonín Ptáček. Writing – review and editing: Marek Jedlička, Jan Válka, and Zdenka Gašová.

## Funding

This study was funded by the Ministry of Health, Czech Republic, which provides institutional support to the research organization number 00023736, the European Regional Development Fund, AIIHHP (Grant CZ.02.1.01/0.0/0.0/16_025/0007428), and OP RDE, MEYS.

## Disclosure

All authors approved the final version of the manuscript and agree to be accountable for all aspects of the work.

## Ethics Statement

All patients included in the study provided their written informed consent to the use of their biological material for research purposes. All experimental procedures involving primary cells were anonymized and followed the ethical standards of the Helsinki Declaration of 1975, as revised in 2008. The experiments were approved by the Ethics Committee of the Institute of Hematology and Blood Transfusion (Prague, Czech Republic).

## Conflicts of Interest

The authors declare no conflicts on interest.

## Supporting Information

Additional supporting information can be found online in the Supporting Information section.

## Supporting information


**Supporting Information 1** Figures S1 to S11 cited in the text are provided in the Supporting Information Figures file.

**Supporting Information 2** Clinical and genetic data of patients with AML is given in the Supporting Information Table S1.

**Supporting Information 3** The WB information file contains raw data from western blot experiments.

## Data Availability

The data that support the findings of this study are available from the corresponding author upon reasonable request.

## References

[1] Brodská B. , Otevřelová P. , Šálek C. , Fuchs O. , Gašová Z. , and Kuželová K. , High PD-L1 Expression Predicts for Worse Outcome of Leukemia Patients With Concomitant NPM1 and FLT3 Mutations, International Journal of Molecular Sciences. (2019) 20, no. 11, 10.3390/ijms20112823, 2823. 31185600 PMC6600137

[2] Toffalori C. , Zito L. , and Gambacorta V. , et al.Immune Signature Drives Leukemia Escape and Relapse After Hematopoietic Cell Transplantation, Nature Medicine. (2019) 25, no. 4, 603–611, 10.1038/s41591-019-0400-z. 30911134

[3] Goltz D. , Gevensleben H. , and Grünen S. , et al.PD-L1 (CD274) Promoter Methylation Predicts Survival in Patients With Acute Myeloid Leukemia, Leukemia. (2017) 31, no. 3, 738–743, 10.1038/leu.2016.328. 27840427

[4] Hargadon K. M. , Genetic Dysregulation of Immunologic and Oncogenic Signaling Pathways Associated With Tumor-Intrinsic Immune Resistance: A Molecular Basis for Combination Targeted Therapy-Immunotherapy for Cancer, Cellular and Molecular Life Sciences. (2023) 80, no. 2, 40–49, 10.1007/s00018-023-04689-9. 36629955 PMC11072992

[5] Austin R. , Smyth M. J. , and Lane S. W. , Harnessing the Immune System in Acute Myeloid Leukaemia, Critical Reviews in Oncology/Hematology. (2016) 103, 62–77, 10.1016/j.critrevonc.2016.04.020. 27247119

[6] Zajac M. , Zaleska J. , Dolnik A. , Bullinger L. , and Giannopoulos K. , Expression of CD274 (PD-L1) Is Associated With Unfavourable Recurrent Mutations in AML, British Journal of Haematology. (2018) 183, no. 5, 822–825, 10.1111/bjh.15040. 29265177

[7] Chai J. , Choudhuri J. , and Wang Q. , et al.Acute Myeloid Leukemias With JAK2/STAT Mutations Are Associated With PD-L1 Upregulation, Leukemia and Lymphoma. (2023) 64, no. 10, 1662–1672, 10.1080/10428194.2023.2232494. 37424335

[8] Berthon C. , Driss V. , and Liu J. , et al.In Acute Myeloid Leukemia, B7-H1 (PD-L1) Protection of Blasts From Cytotoxic T Cells is Induced by TLR Ligands and Interferon-Gamma and can Be Reversed Using MEK Inhibitors, Cancer Immunology, Immunotherapy. (2010) 59, no. 12, 1839–1849, 10.1007/s00262-010-0909-y. 20814675 PMC2945474

[9] Kronig H. , Kremmler L. , and Haller B. , et al.Interferon-Induced Programmed Death-Ligand 1 (PD-L1/B7-H1) Expression Increases on Human Acute Myeloid Leukemia Blast Cells During Treatment, European Journal of Haematology. (2014) 92, no. 3, 195–203, 10.1111/ejh.12228. 24175978

[10] Feng J. , Yang H. , and Zhang Y. , et al.Tumor Cell-Derived Lactate Induces TAZ-Dependent Upregulation of PD-L1 Through GPR81 in Human Lung Cancer Cells, Oncogene. (2017) 36, no. 42, 5829–5839, 10.1038/onc.2017.188. 28604752

[11] Cho S. , Kim W. , and Yoo D. , et al.Impact of Glucose Metabolism on PD-L1 Expression in Sorafenib-Resistant Hepatocellular Carcinoma Cells, Scientific Reports. (2024) 14, no. 1, 10.1038/s41598-024-52160-x, 1751. 38243049 PMC10798953

[12] Morrissey S. M. , Zhang F. , and Ding C. , et al.Tumor-Derived Exosomes Drive Immunosuppressive Macrophages in a Pre-Metastatic Niche Through Glycolytic Dominant Metabolic Reprogramming, Cell Metabolism. (2021) 33, no. 10, 2040–2058.e10, 10.1016/j.cmet.2021.09.002. 34559989 PMC8506837

[13] Yu Y. , Liang Y. , and Li D. , et al.Glucose Metabolism Involved in PD-L1-Mediated Immune Escape in the Malignant Kidney Tumour Microenvironment, Cell Death Discovery. (2021) 7, no. 1, 15–17, 10.1038/s41420-021-00401-7. 33462221 PMC7814120

[14] Carter J. L. , Hege K. , and Kalpage H. A. , et al.Targeting Mitochondrial Respiration for the Treatment of Acute Myeloid Leukemia, Biochemical Pharmacology. (2020) 182, 10.1016/j.bcp.2020.114253, 114253. 33011159 PMC8073742

[15] Lagadinou E. D. , Sach A. , and Callahan K. , et al.BCL-2 Inhibition Targets Oxidative Phosphorylation and Selectively Eradicates Quiescent Human Leukemia Stem Cells, Cell Stem Cell. (2013) 12, no. 3, 329–341, 10.1016/j.stem.2012.12.013. 23333149 PMC3595363

[16] Chen W. L. , Wang J. H. , and Zhao A. H. , et al.A Distinct Glucose Metabolism Signature of Acute Myeloid Leukemia With Prognostic Value, Blood. (2014) 124, no. 10, 1645–1654, 10.1182/blood-2014-02-554204. 25006128 PMC5726328

[17] Catalano G. , Zaza A. , and Banella C. , et al.MCL1 Regulates AML Cells Metabolism via Direct Interaction With HK2. Metabolic Signature at Onset Predicts Overall Survival in AMLs’ Patients, Leukemia. (2023) 37, no. 8, 1600–1610, 10.1038/s41375-023-01946-5. 37349598

[18] Kořánová T. , Dvořáček L. , Grebeňová D. , and Kuželová K. , JR-AB2-011 Induces Fast Metabolic Changes Independent of mTOR Complex 2 Inhibition in Human Leukemia Cells, Pharmacological Reports. (2024) 76, no. 6, 1390–1402, 10.1007/s43440-024-00649-7. 39259491 PMC11582178

[19] Hrdinova T. , Toman O. , and Dresler J. , et al.Exosomes Released by Imatinib-Resistant K562 Cells Contain Specific Membrane Markers, IFITM3, CD146 and CD36 and Increase the Survival of Imatinib-Sensitive Cells in the Presence of Imatinib, International Journal of Oncology. (2020) 58, no. 2, 238–250, 10.3892/ijo.2020.5163. 33491750

[20] Palsson-McDermott E. M. , Dyck L. , and Zaslona Z. , et al.Pyruvate Kinase M2 Is Required for the Expression of the Immune Checkpoint PD-L1 in Immune Cells and Tumors, Frontiers in Immunology. (2017) 8, 10.3389/fimmu.2017.01300, 294695. PMC5646285 29081778

[21] Wang L. , Hückelhoven A. , and Hong J. , et al.Standardization of Cryopreserved Peripheral Blood Mononuclear Cells through a Resting Process for Clinical Immunomonitoring--Development of an Algorithm, Cytometry Part A. (2016) 89, 246–258. 10.1002/cyto.a.22813 26848928

[22] Chiba M. , Shimono J. , and Suto K. , et al.Whole-Genome CRISPR Screening Identifies Molecular Mechanisms of PD-L1 Expression in Adult T-Cell Leukemia/Lymphoma, Blood. (2024) 143, no. 14, 1379–1390, http://ashpublications.org/blood/article-pdf/143/14/1379/2219832/blood_bld-2023-021423-main.pdf, [Accessed August 8, 2024] 10.1182/blood.2023021423. 38142436 PMC11033594

[23] Prestipino A. , Emhardt A. J. , and Aumann K. , et al.Oncogenic JAK2(V617F) Causes PD-L1 Expression, Mediating Immune Escape in Myeloproliferative Neoplasms, Science Translational Medicine. (2018) 10, no. 429, https://pubmed.ncbi.nlm.nih.gov/29467301-https://www.ncbi.nlm.nih.gov/pmc/articles/PMC6034655/, 10.1126/scitranslmed.aam7729. PMC6034655 29467301

[24] Atsaves V. , Tsesmetzis N. , and Chioureas D. , et al.PD-L1 is Commonly Expressed and Transcriptionally Regulated by STAT3 and MYC in ALK-Negative Anaplastic Large-Cell Lymphoma, Leukemia. (2017) 31, no. 7, 1633–1637, http://www.nature.com/leu, 10.1038/leu.2017.103. 28344319

[25] Amaya M. L. , Inguva A. , and Pei S. , et al.The STAT3-MYC Axis Promotes Survival of Leukemia Stem Cells by Regulating SLC1A5 and Oxidative Phosphorylation, Blood. (2022) 139, no. 4, 584–596, 10.1182/blood.2021013201. 34525179 PMC8796651

[26] Yucel B. , Altundağ Kara S. , Cekmen M. B. , Ada S. , and Demircan Tan B. , STAT3 Mediated Regulation of Glucose Metabolism in Leukemia Cells, Gene. (2022) 809, 10.1016/j.gene.2021.146012, 146012. 34655719

[27] Patel S. B. , Nemkov T. , and Stefanoni D. , et al.Metabolic Alterations Mediated by STAT3 Promotes Drug Persistence in CML, Leukemia. (2021) 35, no. 12, 3371–3382, 10.1038/s41375-021-01315-0. 34120146 PMC8632690

[28] Wu B. , Liang Z. , Lan H. , Teng X. , and Wang C. , The Role of PKM2 in Cancer Progression and Its Structural and Biological Basis, Journal of Physiology and Biochemistry. (2024) 80, no. 2, 261–275, 10.1007/s13105-024-01007-0. 38329688

[29] Upadhyay S. , Khan S. , and Hassan M. I. , Exploring the Diverse Role of Pyruvate Kinase M2 in Cancer: Navigating Beyond Glycolysis and the Warburg Effect, Biochimica et Biophysica Acta (BBA)–Reviews on Cancer. (2024) 1879, no. 3, 10.1016/j.bbcan.2024.189089, 189089. 38458358

[30] Shirai T. , Nazarewicz R. R. , and Wallis B. B. , et al.The Glycolytic Enzyme PKM2 Bridges Metabolic and Inflammatory Dysfunction in Coronary Artery Disease, Journal of Experimental Medicine. (2016) 213, no. 3, 337–354, 10.1084/jem.20150900. 26926996 PMC4813677

[31] Huang Z.-W. , Zhang X.-N. , and Zhang L. , et al.STAT5 Promotes PD-L1 Expression by Facilitating Histone Lactylation to Drive Immunosuppression in Acute Myeloid Leukemia, Signal Transduction and Targeted Therapy. (2023) 8, no. 1, 10.1038/s41392-023-01605-2, 391. 37777506 PMC10542808

[32] de Jong M. M. E. , Chen L. , Raaijmakers M. H. G. P. , and Cupedo T. , Bone Marrow Inflammation in Haematological Malignancies, Nature Reviews Immunology. (2024) 24, no. 8, 543–558, 10.1038/s41577-024-01003-x. 38491073

[33] Xie M. , Yu Y. , and Kang R. , et al.PKM2-Dependent Glycolysis Promotes NLRP3 and AIM2 Inflammasome Activation, Nature Communications. (2016) 7, no. 1, 10.1038/ncomms13280, 13280. PMC5093342 27779186

[34] Seo M. , Park M. , and Yook Y. , et al.IL-18 Gene Expression Pattern in Exogenously Treated AML Cells, BMB Reports. (2008) 41, no. 6, 461–465, 10.5483/BMBRep.2008.41.6.461. 18593530

[35] Cha J. H. , Yang W. H. , and Xia W. , et al.Metformin Promotes Antitumor Immunity via Endoplasmic-Reticulum-Associated Degradation of PD-L1, Molecular Cell. (2018) 71, no. 4, 606–620, 10.1016/j.molcel.2018.07.030. 30118680 PMC6786495

[36] Dai X. , Bu X. , and Gao Y. , et al.Energy Status Dictates PD-L1 Protein Abundance and Anti-Tumor Immunity to Enable Checkpoint Blockade, Molecular Cell. (2021) 81, no. 11, 2317–2331, 10.1016/j.molcel.2021.03.037. 33909988 PMC8178223

[37] Ma P. , Xing M. , and Han L. , et al.High PD-L1 Expression Drives Glycolysis via an Akt/mTOR/HIF-1α Axis in Acute Myeloid Leukemia, Oncology Reports. (2020) 43, 999–1009, 10.3892/or.2020.7477. 32020232

[38] Morrissey S. M. and Yan J. , Exosomal PD-L1: Roles in Tumor Progression and Immunotherapy, Trends in Cancer. (2020) 6, no. 7, 550–558, 10.1016/j.trecan.2020.03.002. 32610067 PMC7330176

[39] Akil H. , Bentayeb H. , and Aitamer M. , et al.Analysis of CD20 and PD-L1 Levels on Small Extracellular Vesicles (sEV) Produced by DLBCL Cells and EBV-Transformed B Cells, and Potential Role in T Cell Inhibition, Experimental Hematology & Oncology. (2024) 13, no. 1, 10.1186/s40164-024-00518-2, 53. 38760788 PMC11100054

[40] Brodská B. , Otevřelová P. , and Kuželová K. , Correlation of PD-L1 Surface Expression on Leukemia Cells With the Ratio of PD-L1 mRNA Variants and With Electrophoretic Mobility, Cancer Immunology Research. (2016) 4, no. 10, 815–819, 10.1158/2326-6066.CIR-16-0063. 27543594

[41] Kornepati A. V. R. , Vadlamudi R. K. , and Curiel T. J. , Programmed Death Ligand 1 signals in Cancer Cells, Nature Reviews Cancer. (2022) 22, no. 3, 174–189, 10.1038/s41568-021-00431-4. 35031777 PMC9989967

