# Supplementary material for: PD‐L1 Expression in Acute Myeloid Leukemia Cells: Associations With Cell Metabolism
Source: J Immunol Res. 2026 Jul 1;2026:1427790. doi: 10.1155/jimr/1427790 (PMC13323843; doi:10.1155/jimr/1427790)

PD-L1 expression in acute myeloid leukemia cells: associations with cell metabolism

Supplementary Figures S1-S11

Supplementary Figure S1

Gating strategy for PD-L1 assessment.

A – cell debris was gated out in scattergrams.
B – lymphocytes were gated out in CD45 x SSC dotplots.
C – viable cells were gated as DAPI-negative.
D – singlet events were gated in FSC-A x FSC-H dotplots.
E – cutoff for PD-L1 positivity was set using the FMO control.
F – PD-L1 was measured as the percentage of positive cells (84.2 %) or the difference between the mean fluorescence intensity (MFI) of the sample and MFI of the FMO control.

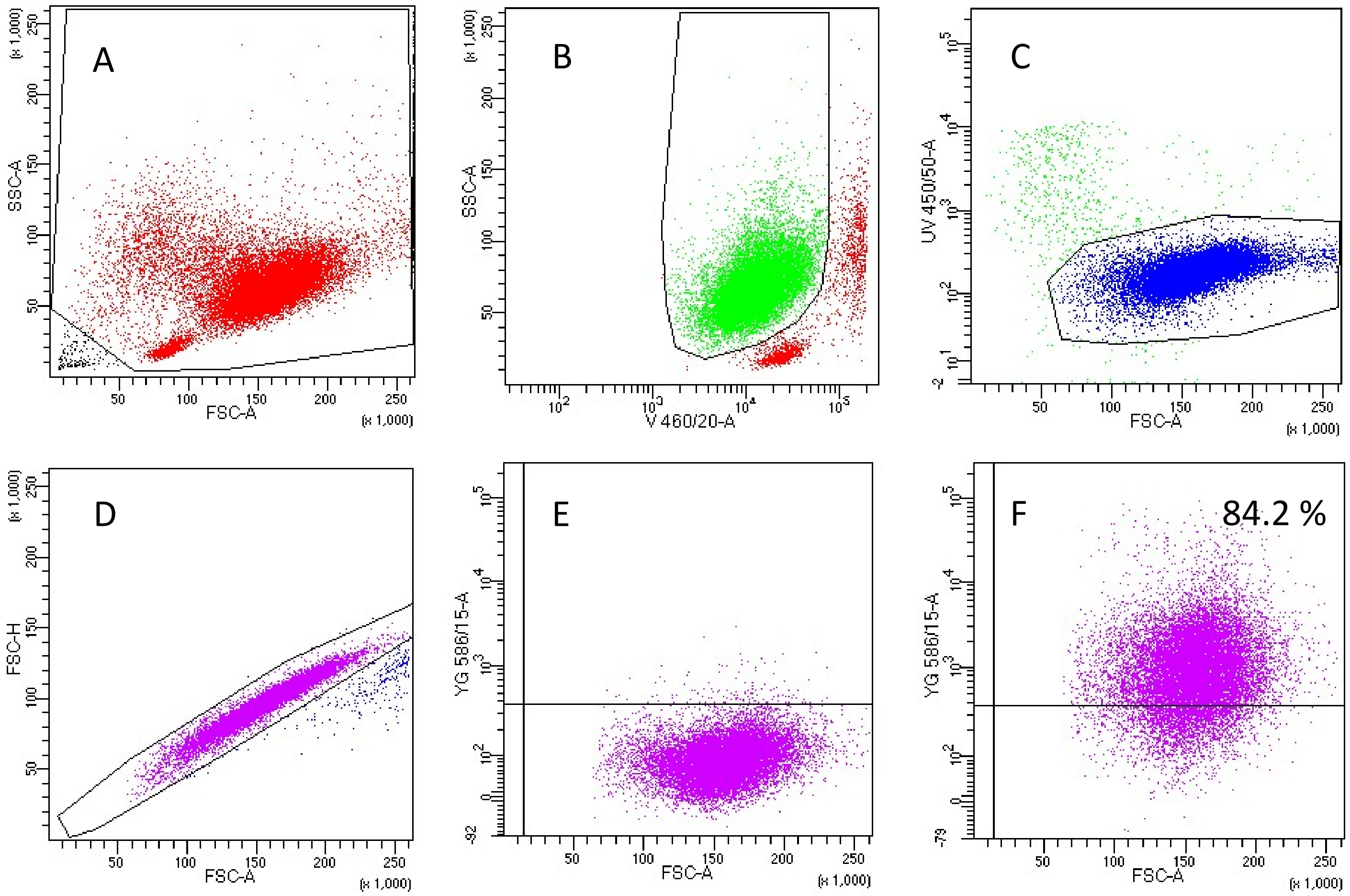

Supplementary Figure S2

This figure complements Fig. 1. Analyses were performed using the values of PD-L1 mean fluorescence intensity (MFI) instead of positive cell fraction.

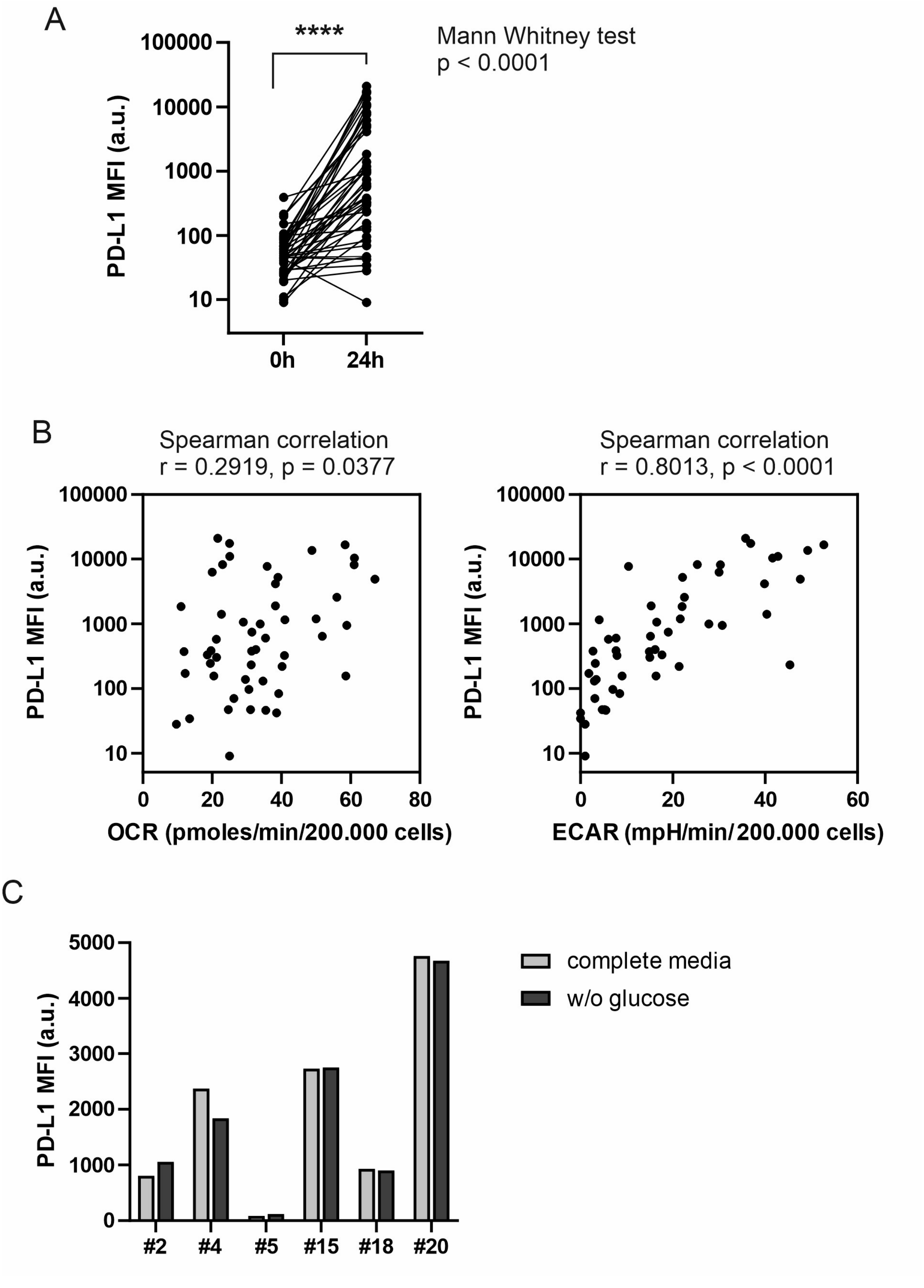

Supplementary Figure S3

PD-L1-positive cell fraction in samples from leukapheresis (LAph, N = 45) or whole peripheral blood (PB, N = 22). In both sample types, fresh isolated peripheral blood mononuclear cells were stained using the same protocol for flow cytometry. The bars show the group medians.

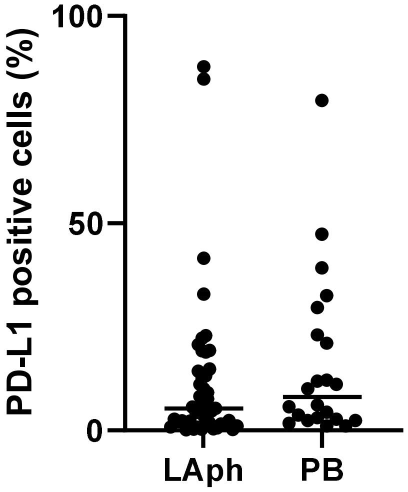

Supplementary Figure S4

Comparison of primary AML cells cultured in media with 10% fetal calf serum (FCS) or 10% human serum (HS, Sigma, H4522-100ML). Cryopreserved aliquots of primary cells from 6 different patients with AML at diagnosis were thawed and cultured overnight in RPMI-1640 with FCS or HS. The rates of cellular respiration (OCR) and glycolysis (ECAR) were analyzed using a Seahorse device (top). Surface PD-L1 was measured by flow cytometry and quantified as the PD-L1-positive cell fraction (left) or mean fluorescence intensity (MFI, right). Differences between samples with FCS and HS were evaluated using the paired Student´s t-test (*p < 0.05).

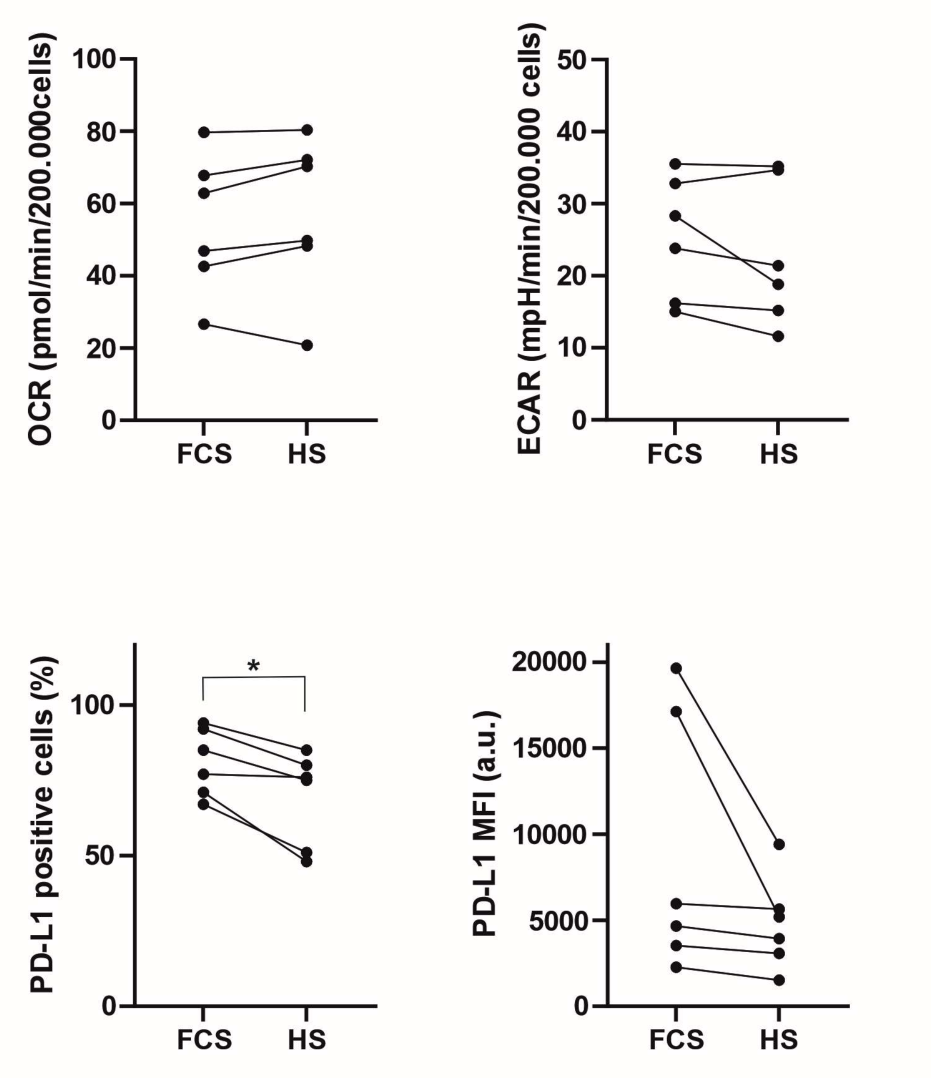

Supplementary Figure S5

Effect of the STAT3 inhibitor stattic on PD-L1 surface expression and cellular metabolism in primary AML cells.
Cryopreserved aliquots of primary AML cells from leukapheresis were thawed and treated overnight with 1 or 5 µM stattic. PD-L1 surface levels (A, N = 9) and metabolic rates (B, N = 7) were measured using flow cytometry and the Seahorse platform, respectively. The differences between groups were assessed using the paired Student´s t-test (*$p < 0.05$, ****$p < 0.0001$).

A

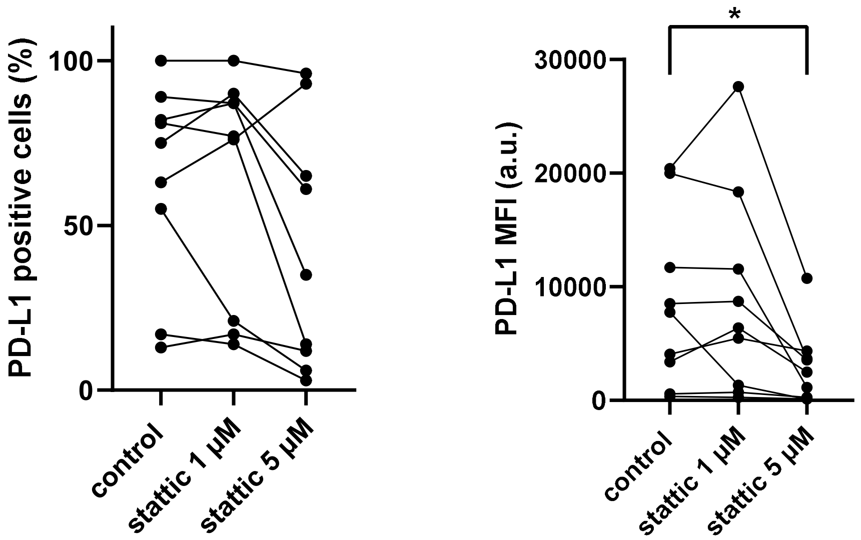

B

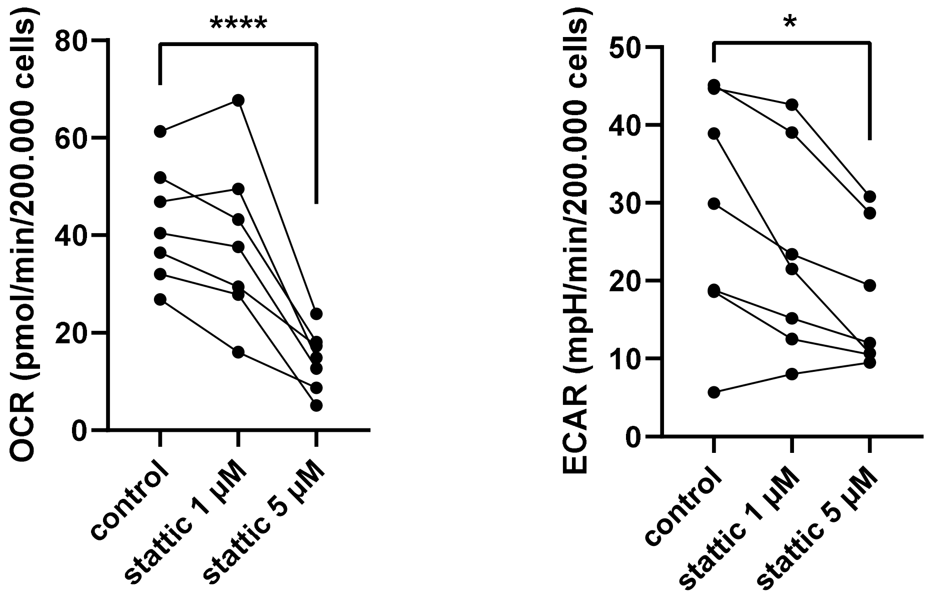

Supplementary Figure S6

Western blot analysis of primary AML cells. Protein extracts from primary AML cells were resolved using SDS electrophoresis and blotted onto a nitrocellulose membrane. Relative intensities of the bands corresponding to the proteins of interests were normalized using β-actin values.

A. Examples of western blot results for PKM2 (summary results in Fig.2A).

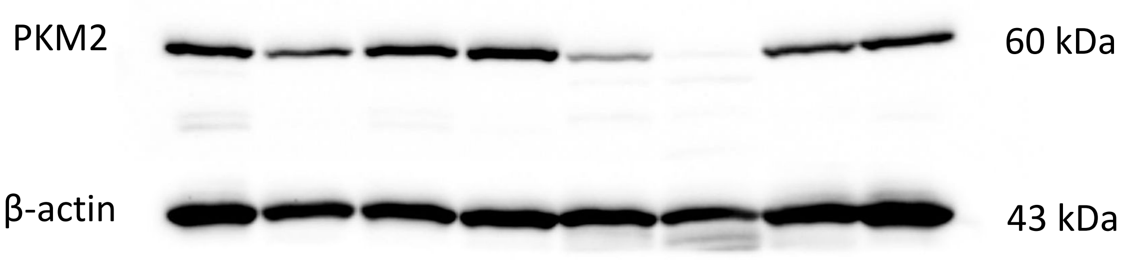

B. pSTAT3 levels in selected primary samples. The assay included 7 samples of patients analyzed in Fig. 2 (B and C), which had rather high PD-L1 levels, and two samples with low PD-L1 expression (the last two lanes). The patient #2 had the JAK2 V617F variant.
Relative band intensities of pSTAT3 normalized to actin are plotted against the fraction of PD-L1 positive cells (left plot) or PD-L1 MFI (right plot).

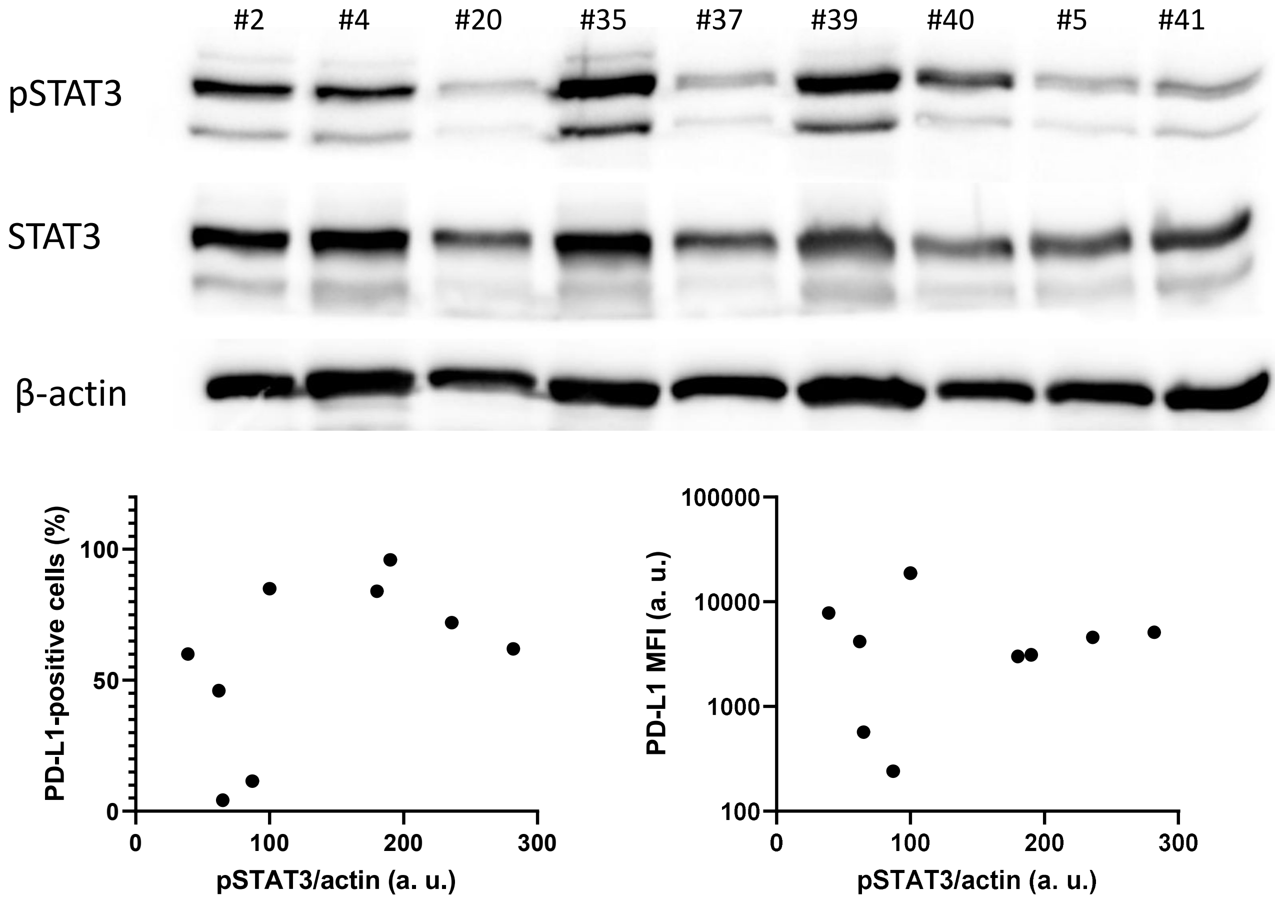

Supplementary Figure S7

A. Effect of PKM2 and JAK1/2 inhibition on pSTAT3 levels.
Examples of western blot membranes and summary results from 6 different primary AML cell samples. The differences between treated samples and controls were evaluated by one sample t-test (reference value = 100).

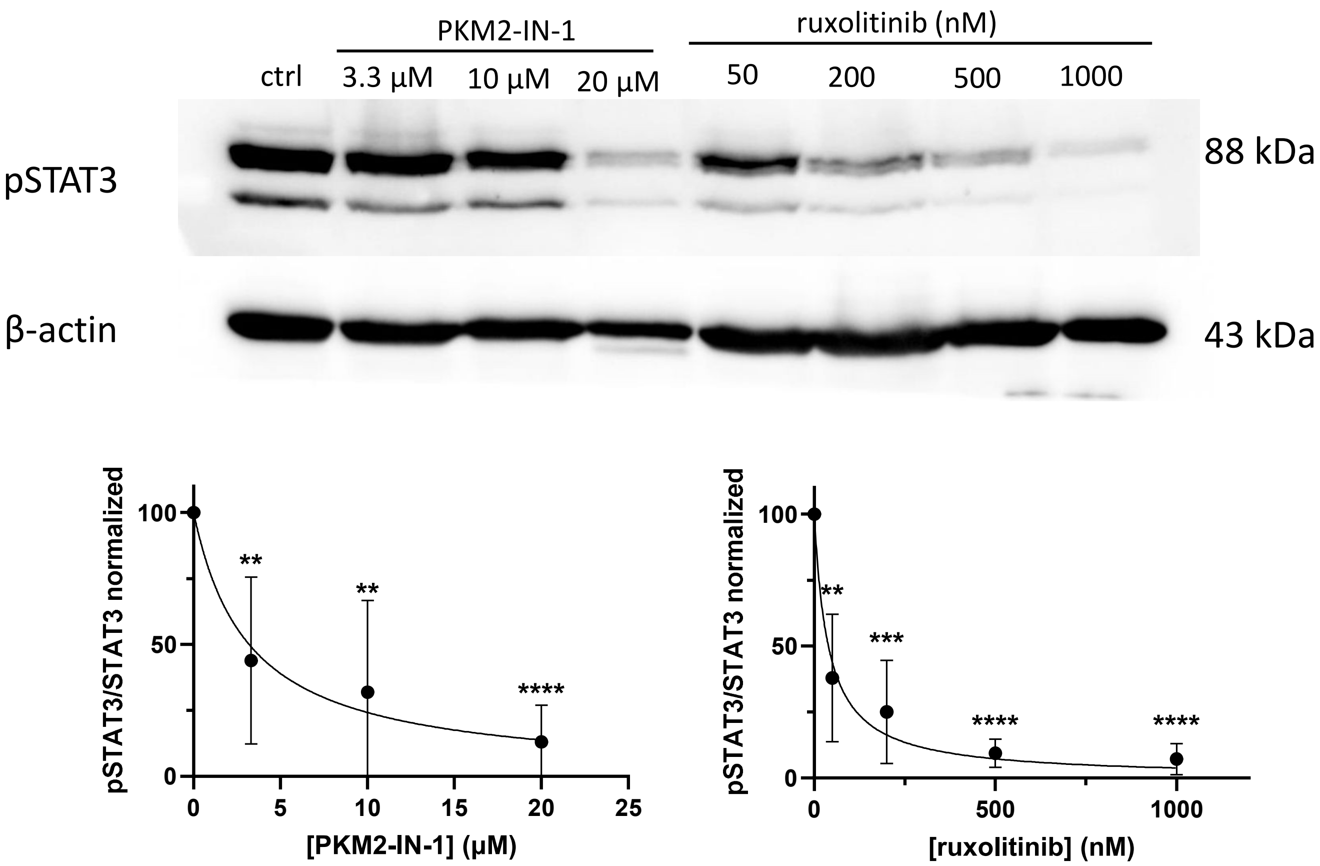

B. Effect of PKM2 and JAK1/2 inhibition in the sample with JAK2 V617F.
One of the samples analyzed in Fig. 2 B+C had JAK2 V617F mutation. The effect of PKM2 or JAK1/2 inhibition on PD-L1 levels for this particular sample is shown here.

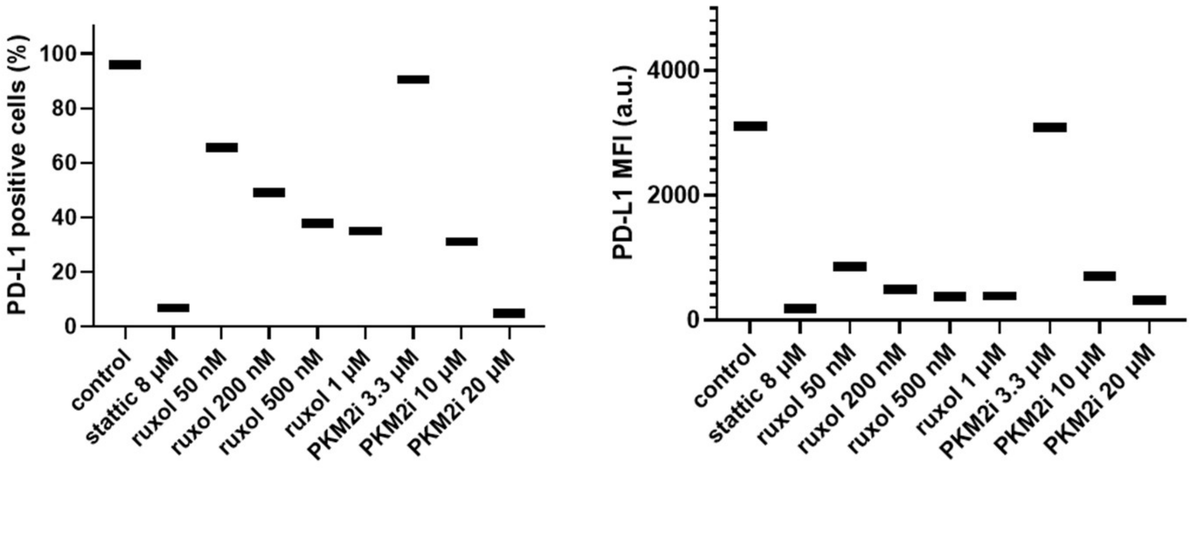

Supplementary Figure S8

Effect of PKM2 inhibition on cellular metabolism in primary AML cells. Cryopreserved aliquots of primary AML cells from leukapheresis (N = 6) were thawed and treated overnight with 10 or 20 µM PKM2-IN-1. The metabolic rates were measured using a Seahorse XFp device. The Seahorse assay medium did not contain pyruvate in this particular series of experiments. The differences between groups were assessed using the paired Student´s t-test (*p < 0.05, **p < 0.01).

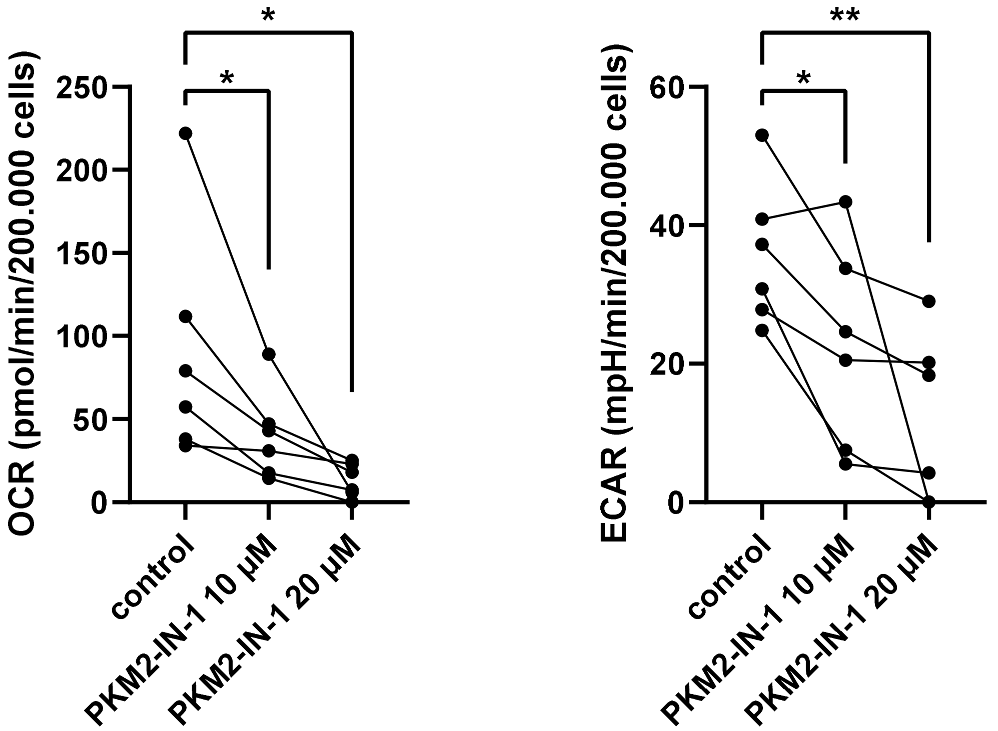

Supplementary Figure S9

Effect of overnight treatment with lactate on PD-L1 surface levels in four AML cell lines as indicated. Means ± SD from 3 experiments for each cell line.

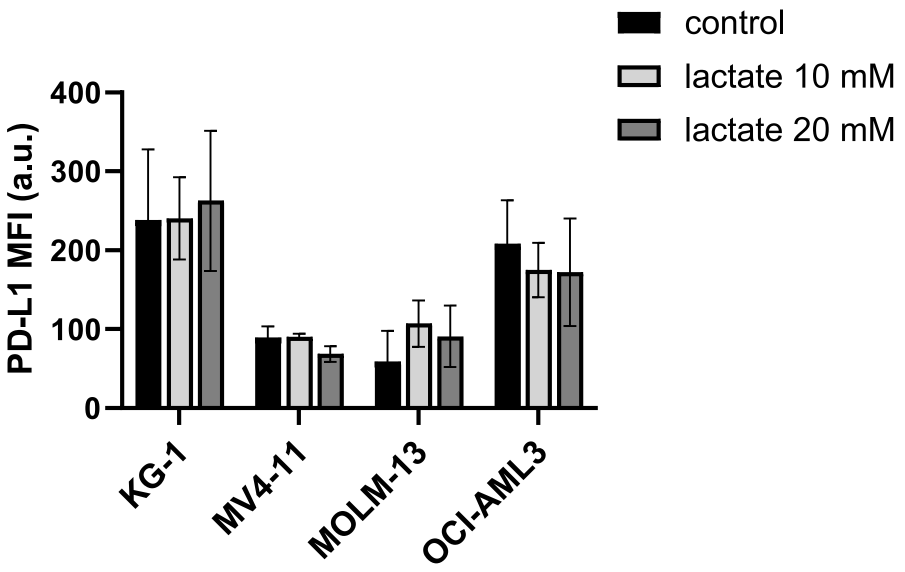

Supplementary Figure S10

Correlation of plasma IL-18 levels with PD-L1 positive cell fraction (left) and PD-L1 MFI (right) of matched primary cells. Results of the Spearman correlation test are given in Table 1.

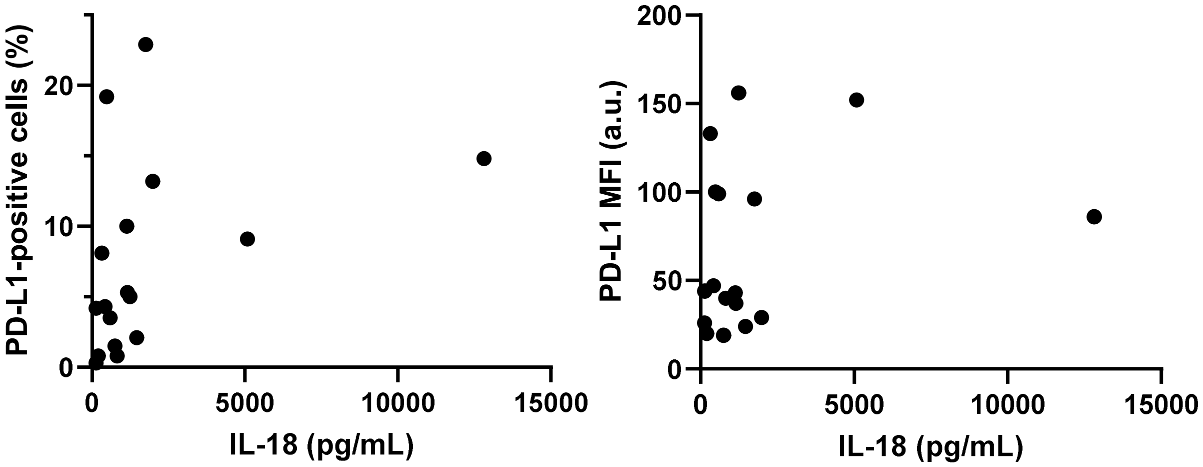

Supplementary Figure S11

Correlation of exosomal PD-L1 with selected measured parameters.
PD-L1 content in exosomes derived from primary AML cells was determined by western blot and normalized to β-actin. The normalized values are plotted against the following parameters found in the matched samples: effect of conditioned media (CM) or primary AML cells on PD-L1 expression on KG-1 cells (top graphs), PD-L1 positivity of the source AML cells (bottom, left), and the glycolysis rate (ECAR) in the source cells (bottom, right). Spearman correlation test was used to assess the possible correlations.

Effect of CM from the source cells on PD-L1 surface levels in KG-1 cells

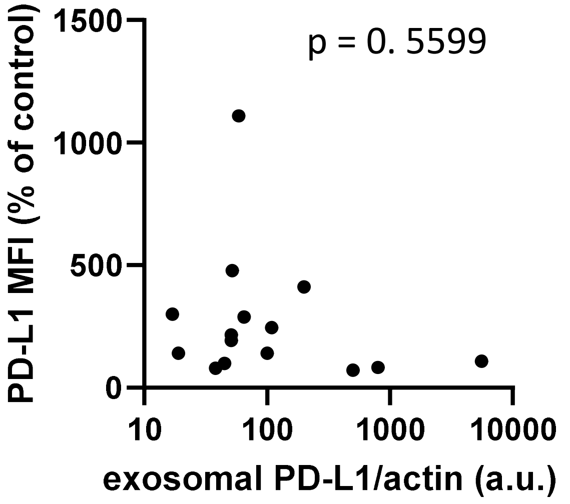

Effect of the source cells on PD-L1 surface levels in KG-1 cells

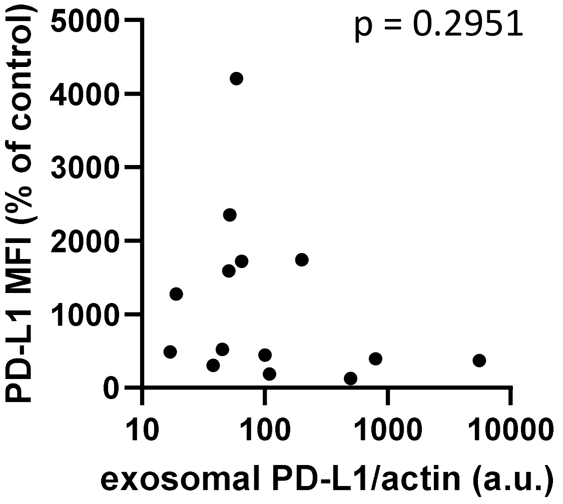

PD-L1 positivity of the source cells

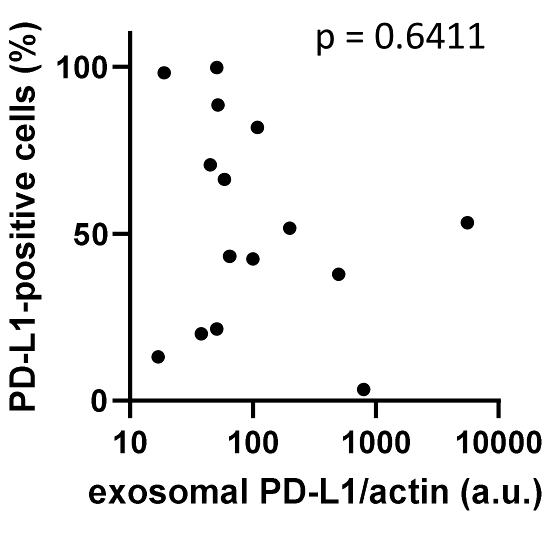

Glycolysis rate in the source cells

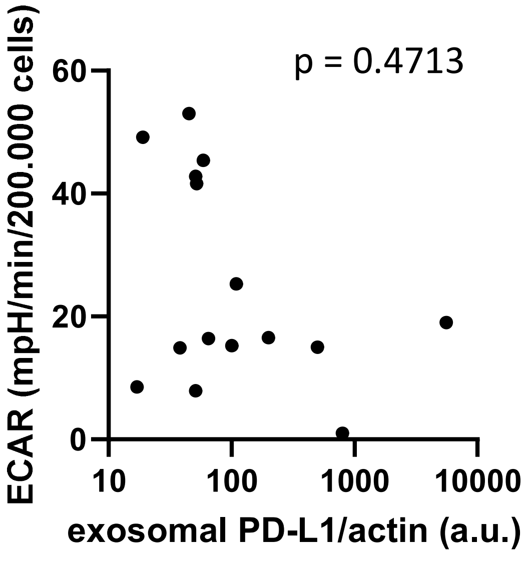

Supplement: Supplementary file 1 — Supporting Information 1 Figures S1 to S11 cited in the text are provided in the Supporting Information Figures file. [file JIMR-2026-1427790-s001.pdf]
