# Supplementary material for: PD‐L1 Expression in Acute Myeloid Leukemia Cells: Associations With Cell Metabolism
Source: J Immunol Res. 2026 Jul 1;2026:1427790. doi: 10.1155/jimr/1427790 (PMC13323843; doi:10.1155/jimr/1427790)

**PD-L1 expression in acute myeloid leukemia cells: associations with cell metabolism**

# Supporting Information

## WB image annotation

Precision Plus Protein™ All Blue Protein Standards (BioRad, #161-0373) or Precision Plus Protein™ Dual Color Protein Standards (BioRad, #161-0374) were used to determine the molecular weight of the western blot bands.

Figure 5B: PD-L1 in source cells

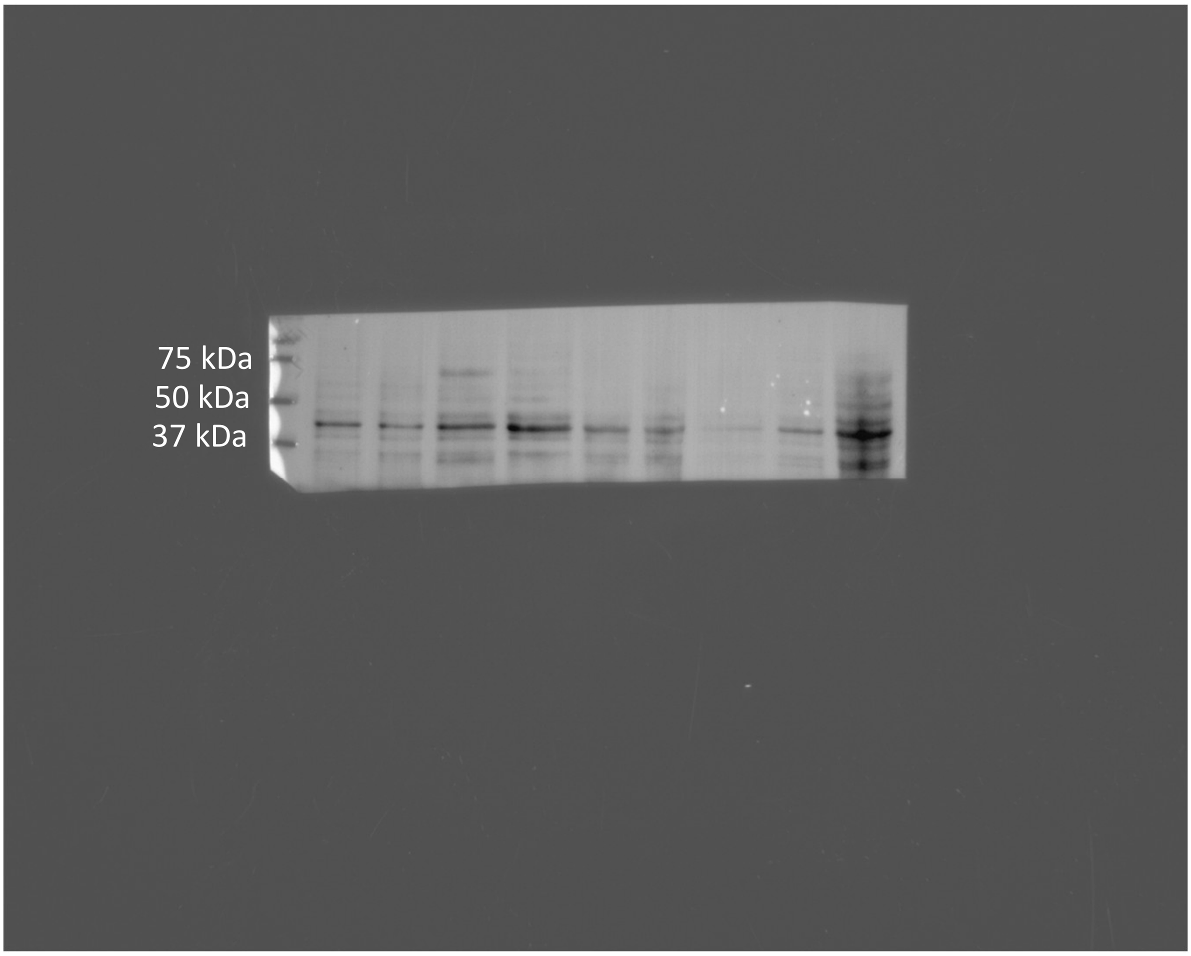

Figure 5B: PD-L1 in exosomes

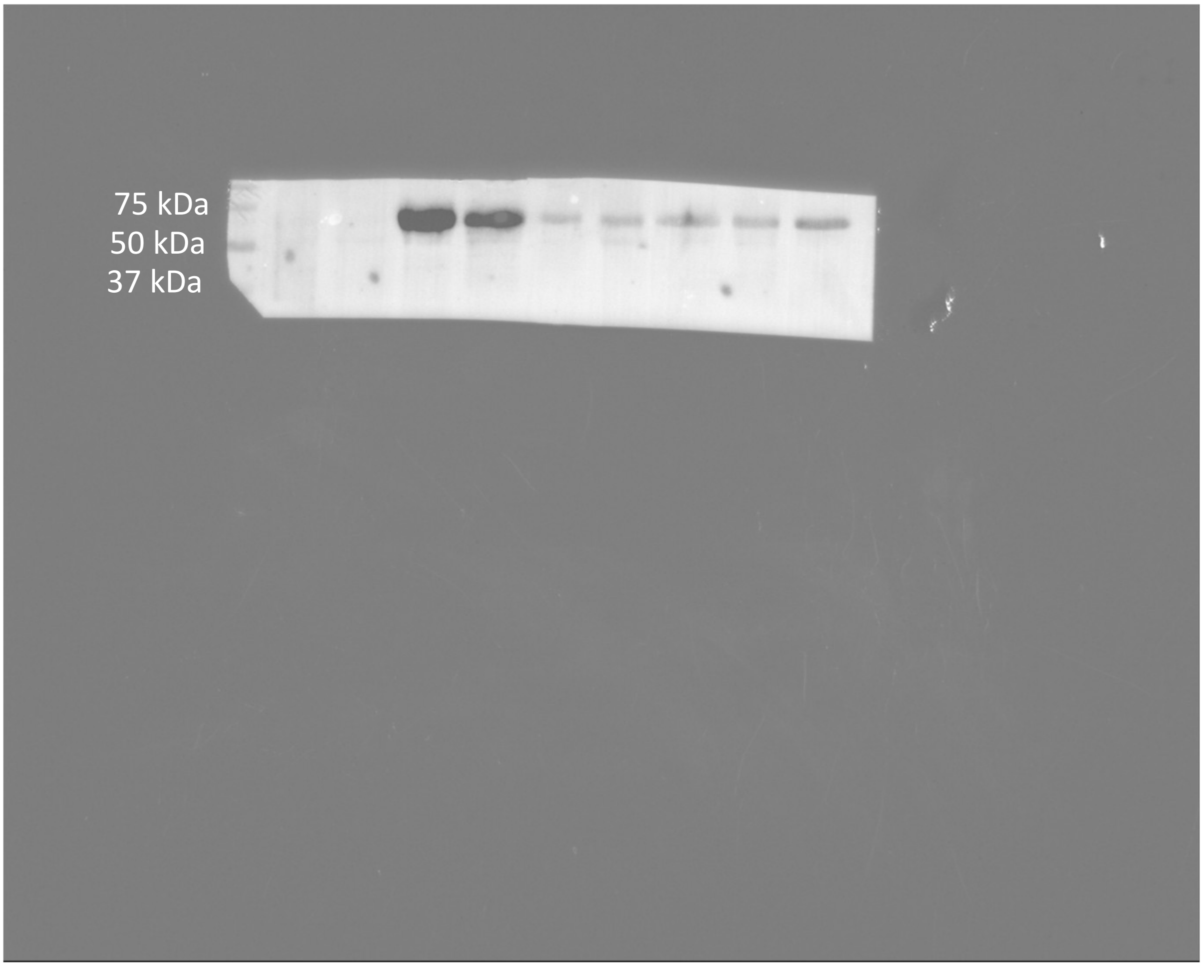

Figure 5B: β-actin in exosomes

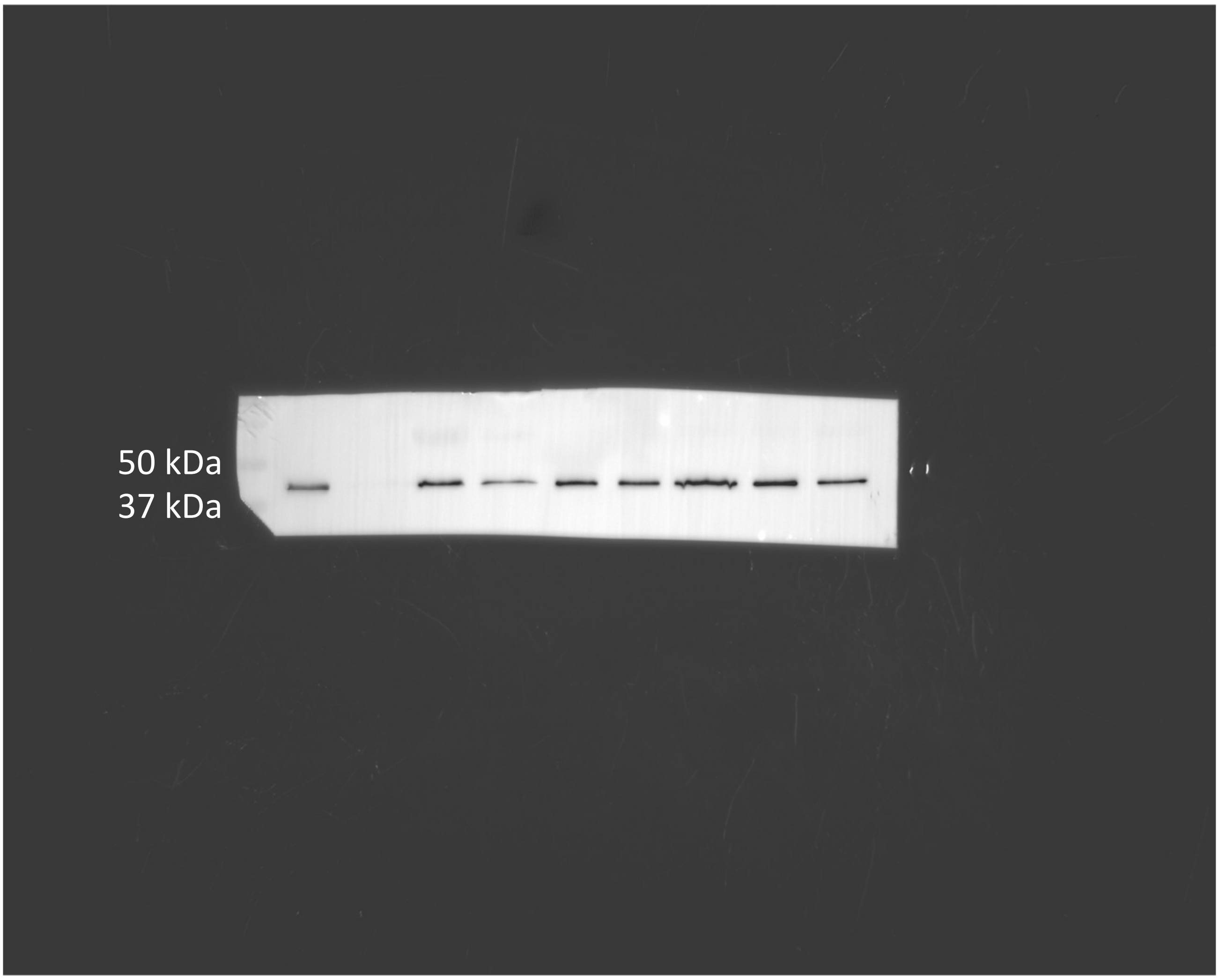

Figure 5B: CD81 in exosomes

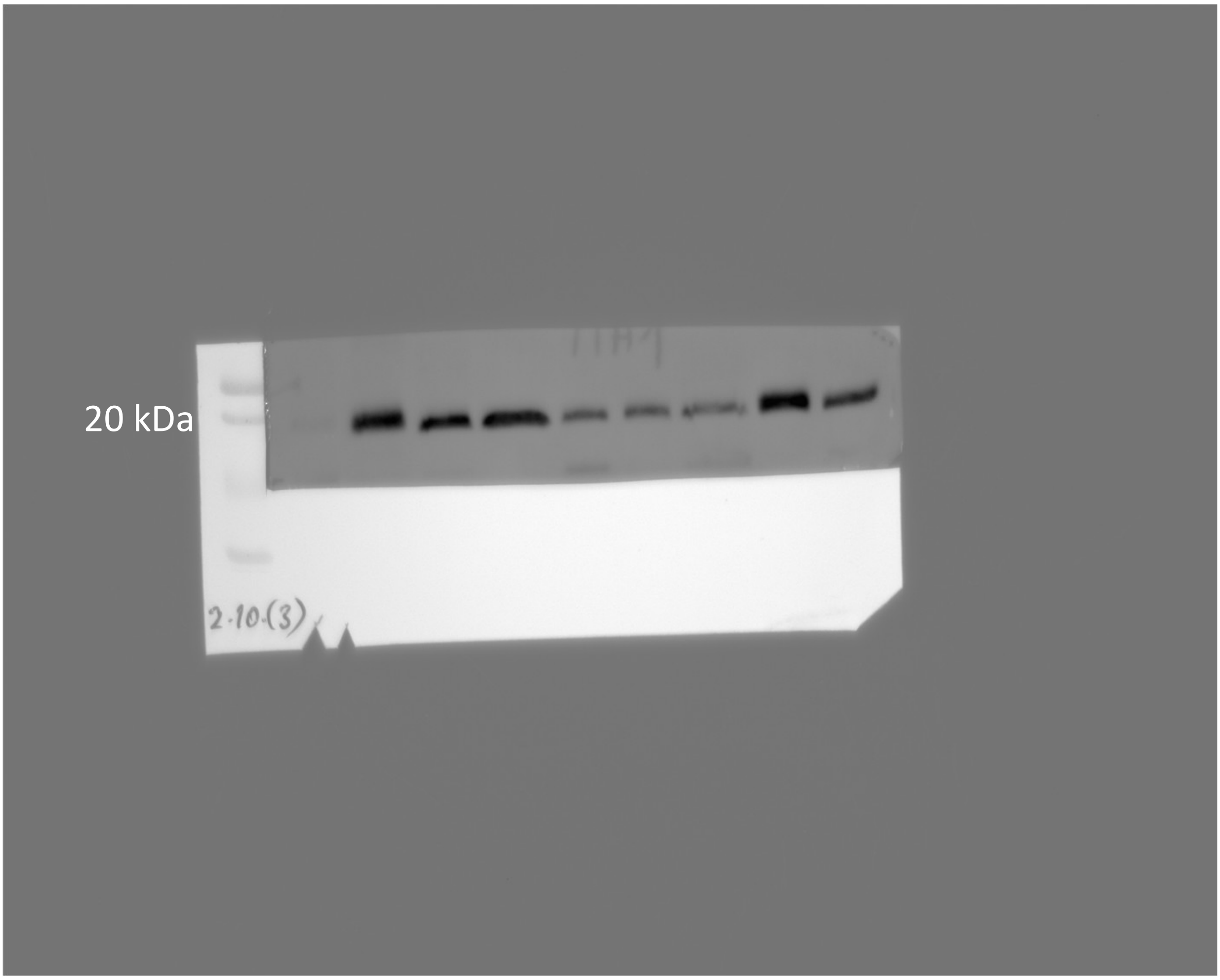

Supplementary Figure S6A: PKM2

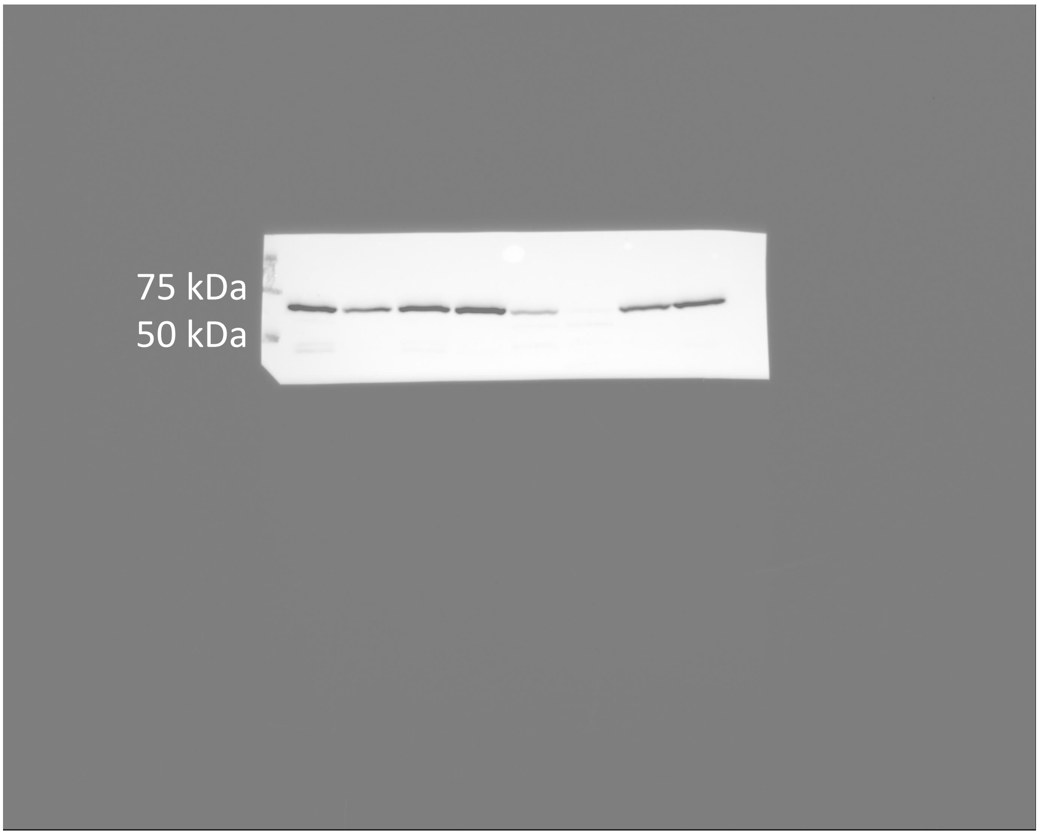


β-actin (reprobed after PKM2)

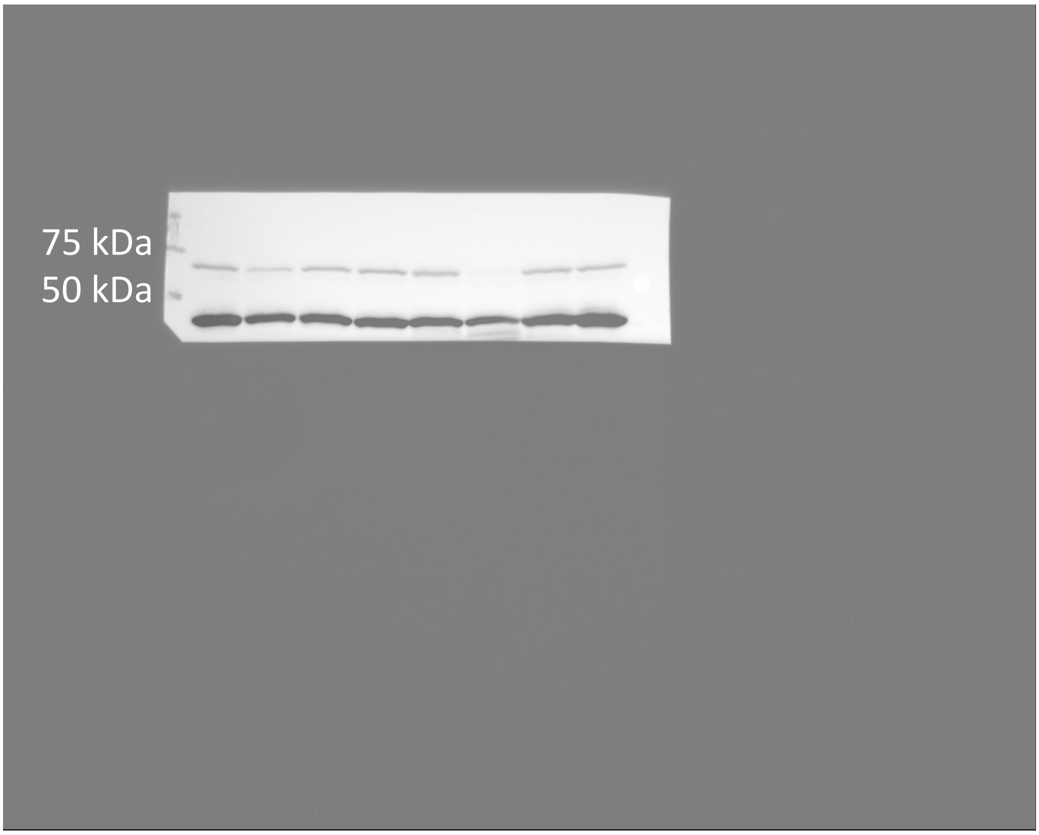

Supplementary Figure S6B: pSTAT3

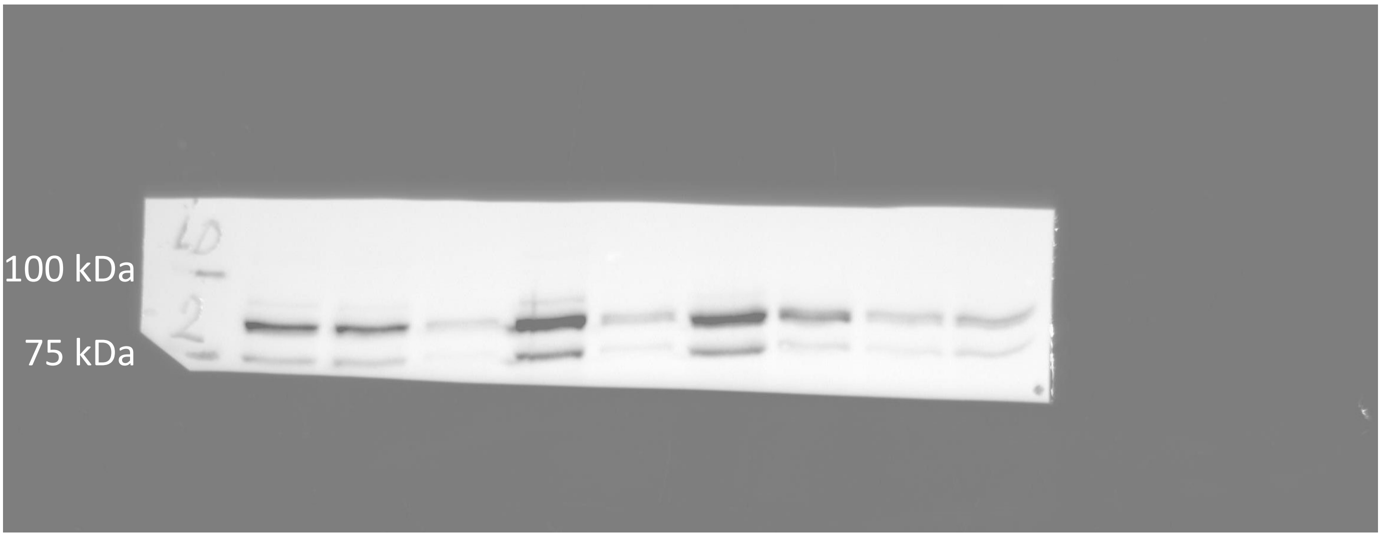


STAT3

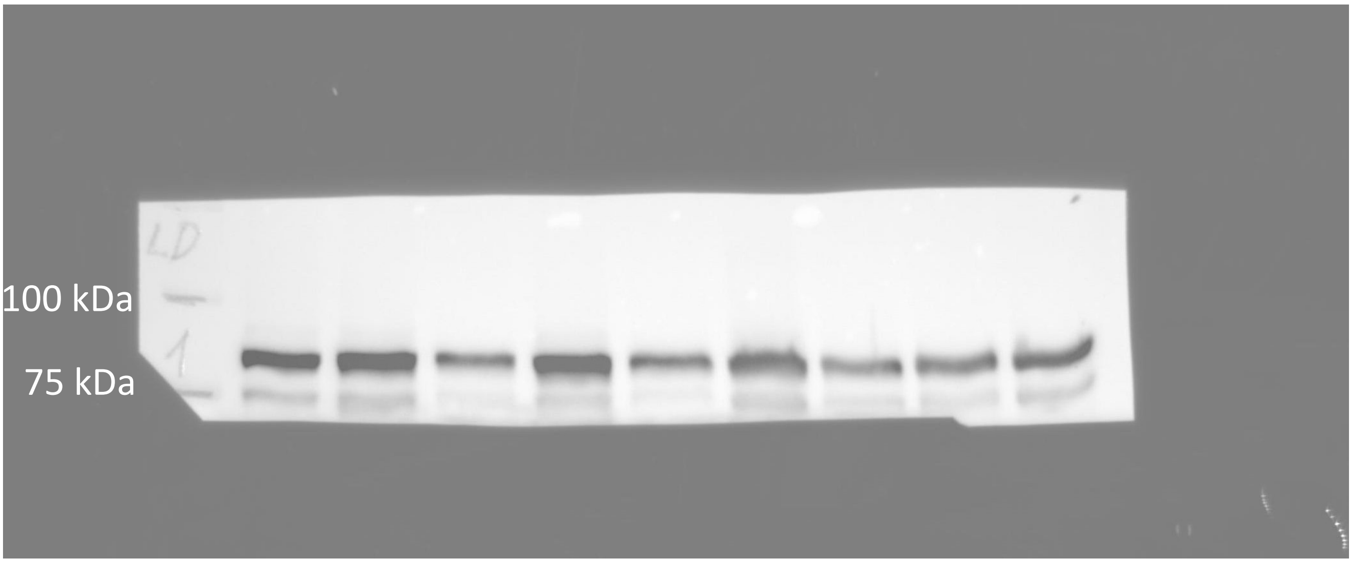


β-actin

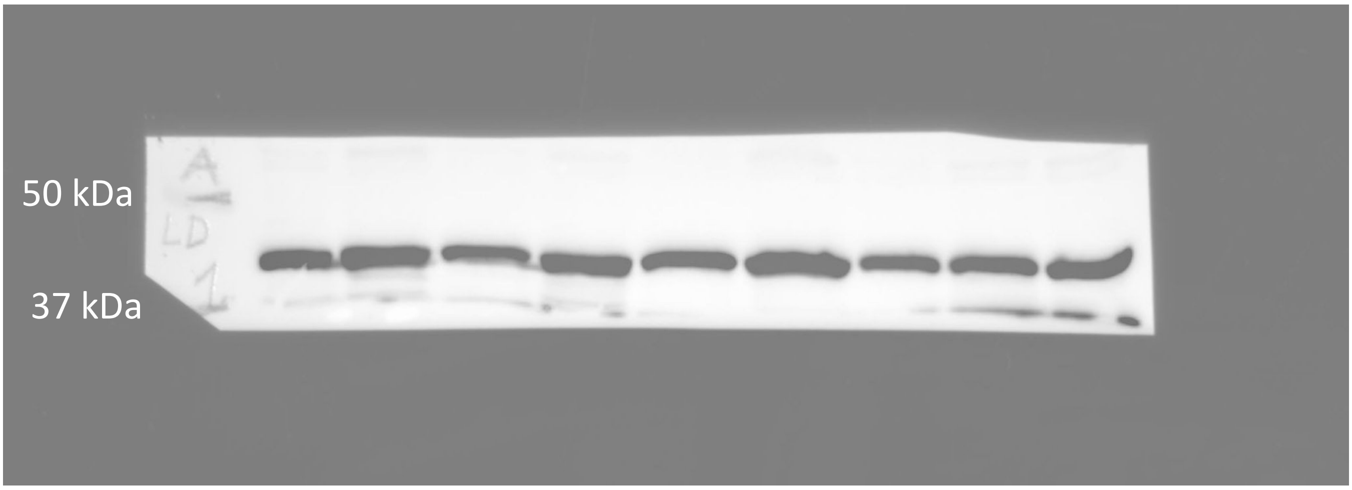

Supplementary Figure S7A: pSTAT3

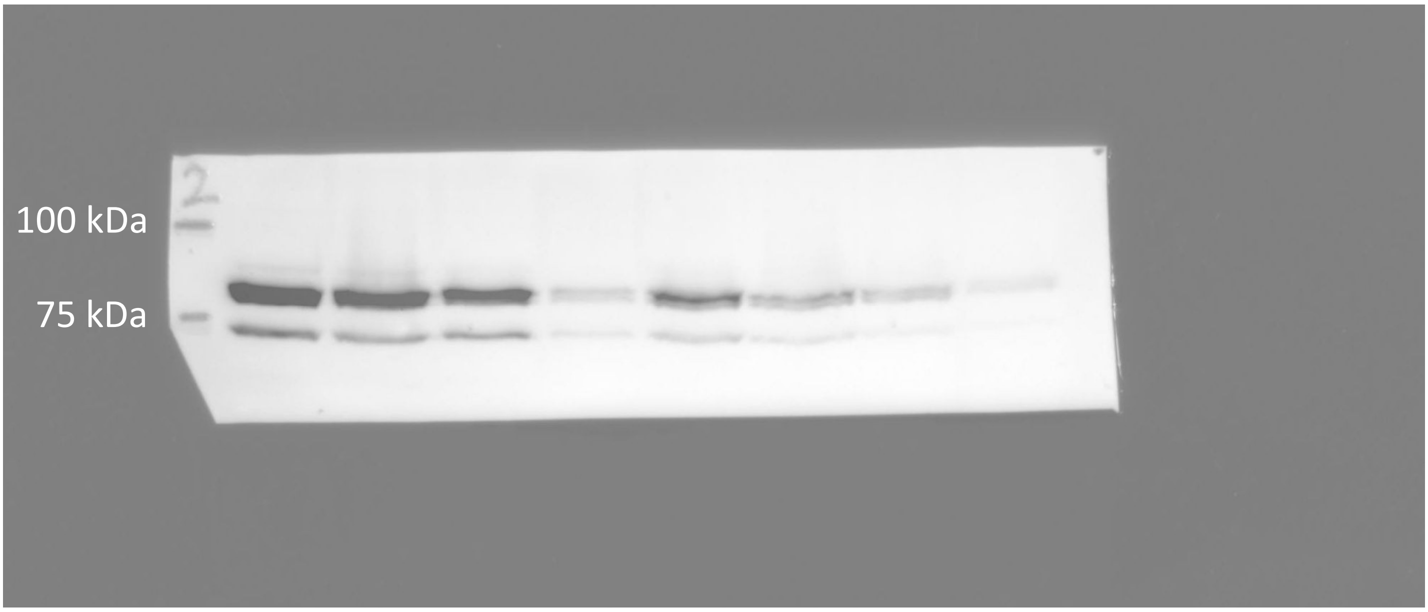


β-actin

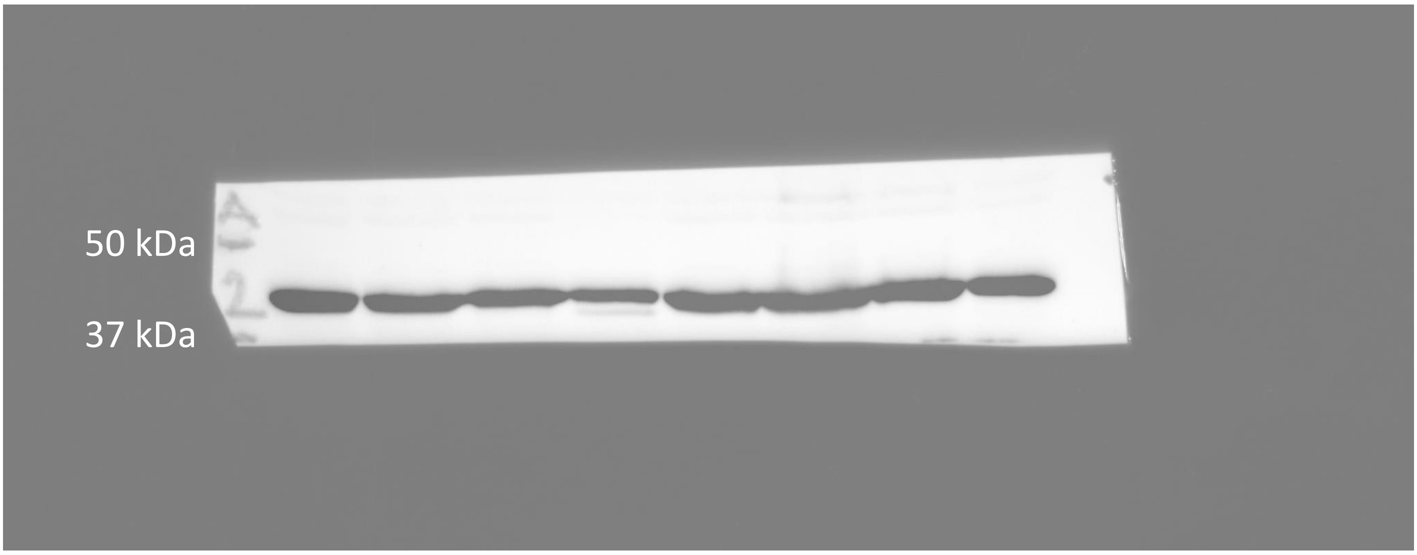

Supplement: Supplementary file 3 — Supporting Information 3 The WB information file contains raw data from western blot experiments. [file JIMR-2026-1427790-s003.pdf]
